# Global, regional and national burden and quality of care index (QCI) of leukaemia and brain and central nervous system tumours in children and adolescents aged 0–19 years: a systematic analysis of the Global Burden of Disease Study 1990–2019

**DOI:** 10.1136/bmjopen-2024-093397

**Published:** 2025-03-22

**Authors:** Yanxin Bi, Kepei Huang, Minmin Wang, Yinzi Jin, Zhi-Jie Zheng

**Affiliations:** 1 Department of Global Health, School of Public Health, Peking University, Beijing, China; 2 Institute for Global Health and Development, Peking University, Beijing, China

**Keywords:** Child, Leukaemia, Health Equity, ONCOLOGY, Paediatric oncology

## Abstract

**Abstract:**

**Objectives:**

This study aimed to evaluate the global, regional and national disparities in the quality of care for leukaemia and brain and central nervous system (CNS) tumours among children and adolescents aged 0–19 years. We also assessed temporal trends in the quality of care index (QCI) and explored associations with sociodemographic development levels, gender and age.

**Setting:**

The study used data from the Global Burden of Disease (GBD) 2019 database, covering 204 countries and territories. The analysis included global, regional and national levels of care, stratified by sociodemographic index (SDI), gender and age groups.

**Participants:**

The study included children and adolescents aged 0–19 years diagnosed with leukaemia or CNS tumours. Data on incidence, prevalence, mortality and disability-adjusted life years (DALYs) were extracted from the GBD 2019 database.

**Primary and secondary outcome measures:**

The primary outcome was the QCI, constructed using principal component analysis from four secondary indicators: years of life lost to years lived with disability ratio, DALYs to prevalence ratio, mortality to incidence ratio and prevalence to incidence ratio. Secondary outcomes included temporal trends in QCI, gender disparity ratios (GDRs) and correlations between QCI and SDI levels.

**Results:**

In 2019, leukaemia and CNS tumours accounted for 132 194 deaths globally. The QCI for leukaemia was 74.71, while for CNS tumours, it was 56.59. From 1990 to 2019, the QCI for CNS tumours increased significantly (estimated annual percentage change (EAPC)=1.45, 95% CI: 1.41 to 1.50), whereas the QCI for leukaemia showed a declining trend in middle and low-middle SDI regions (EAPC=−0.13, 95% CI: −0.16 to –0.09). Western Europe had the highest QCI for leukaemia (94.50), while South Asia had the lowest (57.64). Boys had lower QCI scores than girls, and the gender disparity in CNS tumours widened over time (GDR increased from 1.147 in 1990 to 1.160 in 2019). QCI was positively correlated with SDI levels (leukaemia: r=0.591, p<0.001; CNS tumours: r=0.812, p<0.001).

**Conclusions:**

This study highlights significant disparities in the quality of childhood cancer care across regions, development levels and genders. While global QCI for CNS tumours improved, leukaemia care quality declined in middle and low-middle SDI regions. Boys and populations in low SDI regions are particularly vulnerable to poor care. Policymakers should prioritise targeted interventions to address these disparities, improve access to quality care and reduce the global burden of childhood cancer.

**STRENGTHS AND LIMITATIONS OF THIS STUDY**

- The study used data from the Global Burden of Disease 2019 database, covering 204 countries and territories, providing comprehensive global and regional perspectives.
- A two-step methodology was used to construct the quality of care index (QCI), incorporating principal component analysis to extract and scale indicators.
- Multiple disease burden indicators (incidence, prevalence, mortality, disability adjusted life years, years of life lost, years lived with disability) were employed to assess the quality of childhood cancer care.
- The analysis accounted for disparities by age, gender and sociodemographic index, highlighting differences in QCI across development levels.
- The limitations include the use of secondary data, which restricted the ability to assess disease subtypes or ethnicity separately, potentially influencing the accuracy of the QCI.

## Introduction

 Childhood cancer represents a significant global health challenge, affecting the lives and health of children and adolescents. Defined by the WHO, childhood cancer encompasses cancers diagnosed between birth and 19 years of age, a period that includes both childhood (0–14 years) and adolescence (15–19 years). This age group is often used in global reports to capture a comprehensive understanding of cancer incidence across childhood and adolescence. Among childhood cancers, leukaemia and brain and central nervous system (CNS) tumours are the most prevalent types.^1^ Nearly 400 000 children aged 0–19 years develop cancer each year globally,^2^ and 13.7 million cases will be diagnosed and 11.1 million children will die of childhood cancer in the next 30 years, posing a huge challenge to children’s health and social development.^3^ In response, WHO launched the global childhood cancer initiative in 2018, aiming to achieve at least 60% survival for childhood cancers worldwide by 2030, thereby saving one million children with cancer over the next decade.^4^

Large geographic inequality existed across regions and countries regarding the disease burden as well as the prognosis of childhood cancer, and the disparity in childhood cancer care quality was assumed to be the reason behind this. For example, the 5-year survival rate of childhood cancer reached 80% in high-income countries (HICs); however, the survival rate was only 15% to 45% in low- and middle-income countries (LMICs) even though LMICs accounted for 90% of global childhood cancer patients.^5^ Quality of care is crucial to improve the outcome and reduce disease burden, especially for childhood cancer due to unclear carcinogenesis mechanisms as well as limited prevention measures.

The quality of care index (QCI) has been a widely applied indicator to measure the cancer care quality at the country, regional and global levels, and previous studies have demonstrated that QCI is strongly associated with tumour prognosis and disease burden.^6^,^8^ However, the indicators as well as the measurement of quality of care for childhood cancer were limited, and neither had evaluated the disparity in childhood cancer care quality across regions, counties and social development levels.

In this study, we present the development and application of the QCI to measure and evaluate disparities in the quality of childhood cancer care across geographic regions, development levels, genders and age groups. Our findings provide essential insights into the populations most vulnerable to poor care, which is critical for informing targeted interventions in childhood cancer prevention and treatment. By examining these disparities, we aim to contribute to the global initiative and help guide efforts to reduce childhood cancer burden and improve outcomes.

## Materials and methods

### Data resources

Data on the disease burden of leukaemia and CNS tumours in children and adolescents aged 0–19 years were obtained from the Global Health Data Exchange (GHDx, https://ghdx.healthdata.org) and Global Burden of Diseases, Injuries, and Risk Factors Study (GBD) 2019. The GBD dataset was established through a systematic approach to global, national and other categories of countries and regions to describe epidemiological data on various diseases, risk factors and injuries stratified by sex, age and geographical categories from 2002. The GBD 2019 incorporated nationally representative surveys, censuses and meta-analysis results to estimate the incidence, prevalence, mortality, years of life lost (YLLs), years lived with disability (YLDs) and disability adjusted life years (DALYs) for 369 diseases and injuries in 204 countries and territories.

In this study, six indicators (incidence, prevalence, mortality, DALYs, YLLs and YLDs) of leukaemia and CNS tumours were collected from the GHDx dataset. Leukaemia was defined as C91–C91.0, C91.2–C91.3, C91.6, C92–C92.6, C93–C93.1, C93.3, C93.8, C94–C95.9 according to the 10th revision of the International Classification of Diseases system, and CNS tumours were defined as malignant neoplasm of meninges, brains and C70–C72.9. Age-standardised measurements were reported per 100 000 persons.

### Quality of care index

The QCI was constructed for leukaemia and CNS tumours to represent the quality of childhood cancer care using the typical methodology applied in previous studies.^8 9^ The construction of the QCI followed a two-step procedure. First, four secondary indicators from six primary parameters were calculated, that is, (1) ratio of YLLs to YLDs ratio, (2) DALYs to prevalence ratio, (3) mortality to incidence ratio and (4) prevalence to incidence ratio.^10^

$$Ratio\;of\;YLLs\;and\;YLDs=\frac{YLLs}{YLDs}$$

$$Ratio\;of\;DALYs\;to\;prevalence=\frac{DALYs}{Prevalence}$$

$$Mortality-\;to-\;incidence\;ratio=\frac{Mortality}{Incidence}$$

$$Prevalence-to-incidence\;ratio=\frac{Prevalence}{Incidence}$$

Second, principal component analysis (PCA) performed on the four secondary indicators to extract the first principal component, which explained the majority of the variance across regions. This component was then scaled to a 0–100 range to produce the final QCI score, where higher values indicated better quality of care. The rationale for PCA was its ability to reduce dimensionality and combine correlated indicators into a single, interpretable metric. The detailed calculation procedure has been described in previous studies.^9 10^

### Statistical analysis

Estimated annual percentage change (EAPC) was calculated to quantify the temporal trend of the QCI by applying a generalised linear model based on Gaussian distribution. For this analysis, a generalised linear regression model was applied to the natural logarithm of age-standardised ratios (ASRs) for QCI, YLLs, YLDs and other relevant metrics. The regression model took the form ln(ASR)=α+β·Year+ε, where α represents the intercept, β represents the positive or negative ASR trends and ε is the error term. The EAPC was derived using the formula 100×(exp[β]−1) and its 95% CIs were obtained directly from the regression coefficients. A positive EAPC value indicated an increasing trend, whereas a negative EAPC signified a decreasing trend over the study period. An increasing temporal trend was identified with an EAPC>0, and a decreasing trend was identified with an EAPC<0.

Correlations between QCI and the sociodemographic index (SDI) were assessed using Pearson correlation coefficients to examine whether higher levels of socioeconomic development were associated with better quality of care. SDI is a composite indicator of a country’s lag-distributed income per capita, average years of schooling and the fertility rate in females under the age of 25 years.^11^ The SDI indicator was also extracted from the GBD 2019 data set (https://ghdx.healthdata.org/record/ihme-data/gbd-2019-socio-demographic-index-sdi1950-2019). In 2019, countries and regions were divided into five levels: high (0.81–1.00), high-middle (0.70–0.81), middle (0.61–0.69), low-middle (0.46–0.60) and low (0.00–0.45).^12^ Additionally, disparities in QCI across sex and age groups were investigated. For gender disparity, the gender disparity ratio (GDR) was calculated as the ratio of QCI in girls divided by the QCI score in boys, where GDR>1 represented a better QCI level in girls than boys. The QCI score was also analysed across five age groups (<1 year, 1–4 years, 5–9 years, 10–14 years and 15–19 years) to identify age-related trends.

$$GDR=\frac{QCIofgirls}{QCIofboys}$$

Uncertainty intervals (UIs) for all GBD-derived estimates were calculated using standardised GBD methods, which incorporate sampling variability, non-sampling error and model uncertainty. These intervals were used to determine statistical significance, defined as non-overlapping UIs between comparison groups. All statistical analyses in this study were conducted using Stata MP (V.18.0; Stata Corp LLC). All tests were two-sided and p values<0.05 were considered statistically significant.

## Results

### Disease burden of childhood cancer

**Figure 1:**
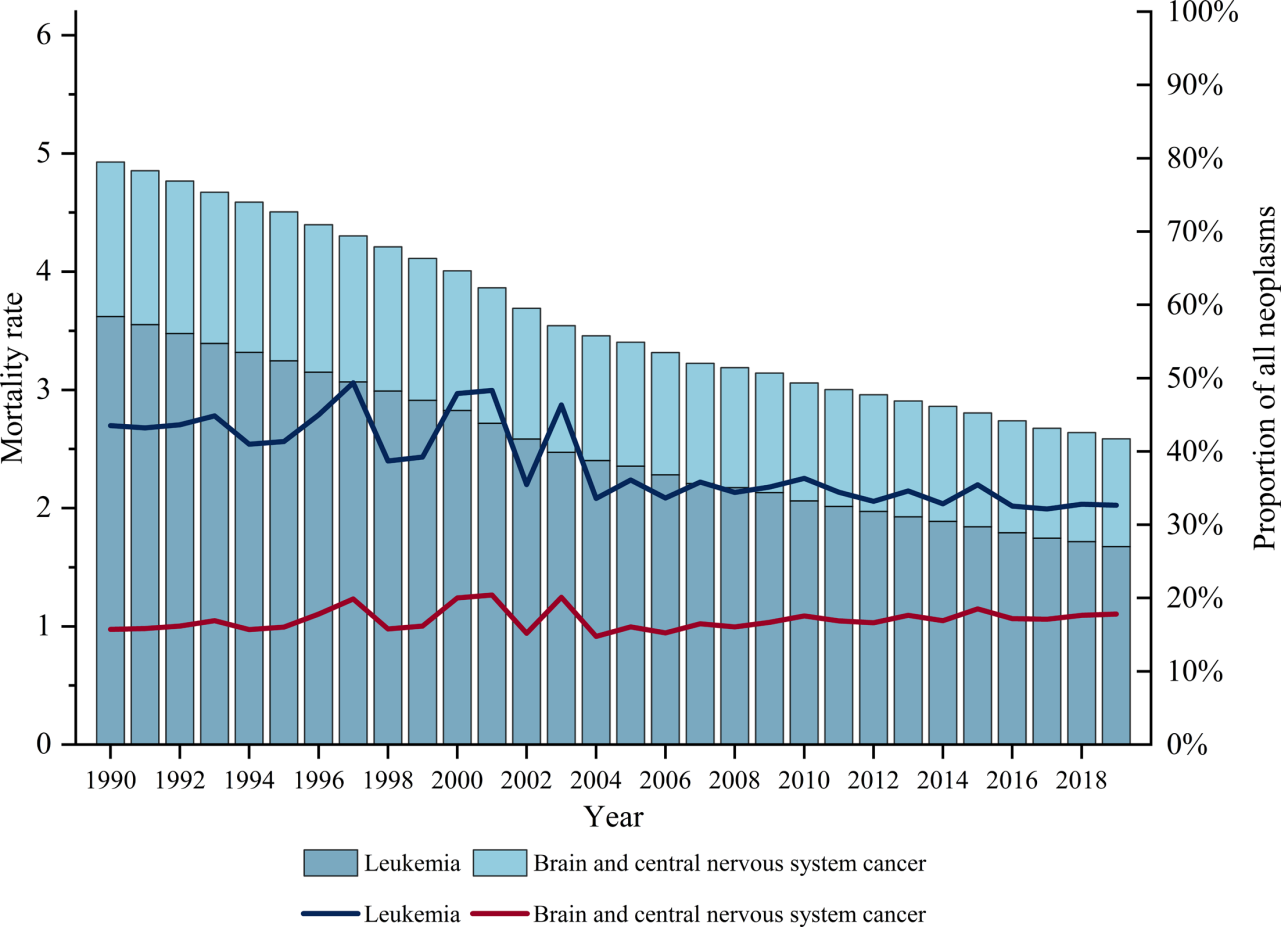
Age-standardised mortality rate and proportion of leukaemia and brain and central nervous system (CNS) tumours, from 1990 to 2019. The histogram showed the age-standardised mortality rate of leukaemia and CNS tumours (per 100 000 population) at global level from 1990 to 2019. The line chart suggested the deaths rate of leukaemia and CNS tumour out of the total deaths.

In 2019, childhood neoplasms caused 10 752 050.79 DALYs (95% UI: 12 163 650.21 to 9 524 498.86), with a total of 132 194 deaths. Leukaemia and brain and CNS tumours are the two most prevalent malignant cancers in children. As shown in figure 1, deaths due to leukaemia decreased from 82 302 to 43 193, with an age-standardised rate decreasing from 3.62 (95% UI: 4.74 to 2.74) to 1.67 (95% UI: 1.91 to 1.45) per 100 000 person-years. Similarly, deaths due to brain and CNS tumours decreased from 29 735 to 23,538, with the age-standardised rate decreasing from 1.31 (95% UI: 2.09 to 0.87) to 0.91 (95% UI: 1.07 to 0.70) per 100 000 person-years (online supplemental table 1). The proportion of deaths from CNS tumour out of total childhood cancer deaths increased from 15.73% in 1990 to 17.81% in 2019, while the proportion of leukaemia deaths fluctuated from 43.53 in 1990 to 46.34 in 2003, before decreasing thereafter.

### Disparity of QCI across geographic regions

In 2019, the estimated QCI for leukaemia was 74.71 and the QCI of brain and CNS tumours was 56.59. From 1990 to 2019, the QCI of brain and CNS tumour displayed an increasing temporal trend with an EAPC of 1.45 (95% CI: 1.41 to 1.50), while Central Sub-Saharan Africa showed the smallest increase with an EAPC of 0.12 (95% CI: 0.06 to 0.19) from 1990 to 2019 (online supplemental table 2).

The QCI of leukaemia and brain and CNS tumours was of disparity across geographic regions and countries. In 2019, Western Europe had the highest QCI for leukaemia (94.50), South Asia had the lowest QCI (57.64); for brain and CNS tumours, high-income Asia Pacific and Central Sub-Saharan Africa had the highest and lowest QCI, respectively. From 1990 to 2019, Eastern Europe was of the highest growing trend of QCI for leukaemia with an EAPC of 0.79 (95 CI%: 0.59 to 0.98), while Central Latin America was of the largest decreasing trend of QCI for leukaemia with an EAPC of −0.41 (95 CI%: −0.45 to –0.38). For CNS tumour, East Asia was of the highest growing trend of QCI with an EAPC of 2.93 (95 CI%: 2.78 to 3.08), while Oceania was of the largest decreasing trend of QCI with an EAPC of −0.03 (95 CI%: −0.11 to 0.05) from 1990 to 2019 (online supplemental table 2).

**Figure 2:**
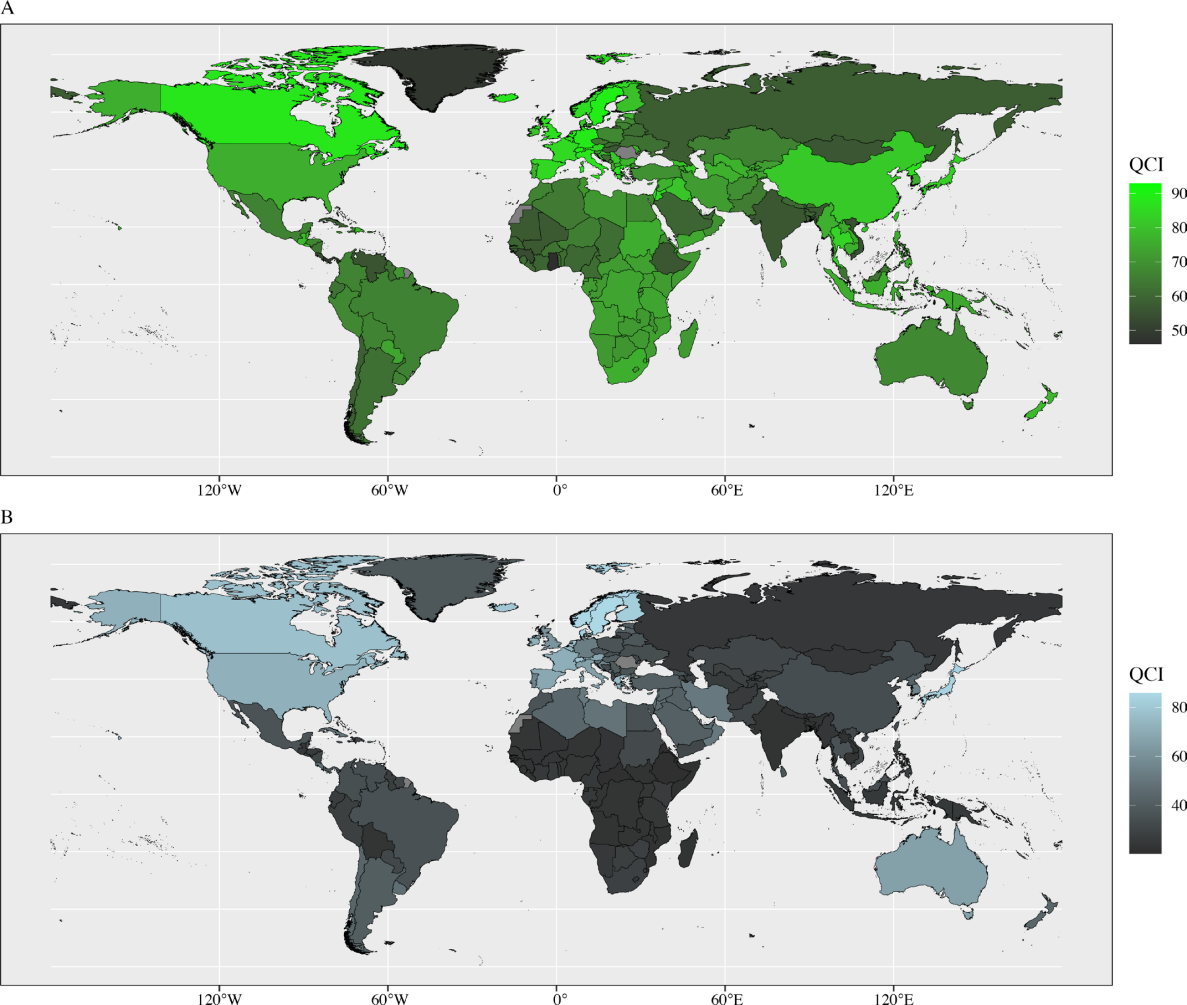
Global map of quality of care index (QCI) for leukaemia and brain and central nervous system (CNS) tumours in 2019. (A) Global map of QCI for leukaemia in 204 countries and territories in 2019. (B) Global map of QCI for CNS tumour in 204 countries and territories in 2019. The QCI was calculated based on principal component analysis, and a higher QCI represented good quality of cancer care.

The distribution of the two childhood cancers’ QCI among different countries in 2019 is as shown in figure 2. At the country level, San Marino had the highest QCI for leukaemia (97.07), Ghana had the lowest QCI (48.71); for CNS tumours, Denmark and the Central African Republic had the highest and lowest QCI, respectively. From 1990 to 2019, Hungary showed the highest growing trend of the QCI (EAPC=1.12, 95% CI: 0.99 to 1.24) and Kyrgyzstan showed the largest decreasing trend of the QCI (EAPC=−0.77, 95% CI: −0.84 to –0.71) for leukaemia; China showed the highest growing trend of the QCI (EAPC=3.02, 95% CI: 2.87 to 3.18) and Zimbabwe showed the largest decreasing trend of the QCI (EAPC=−0.99, 95% CI: −1.20 to –0.78) for brain and CNS tumours (online supplemental table 2 and online supplemental figure 1).

### Disparity of QCI across social development levels

**Figure 3:**
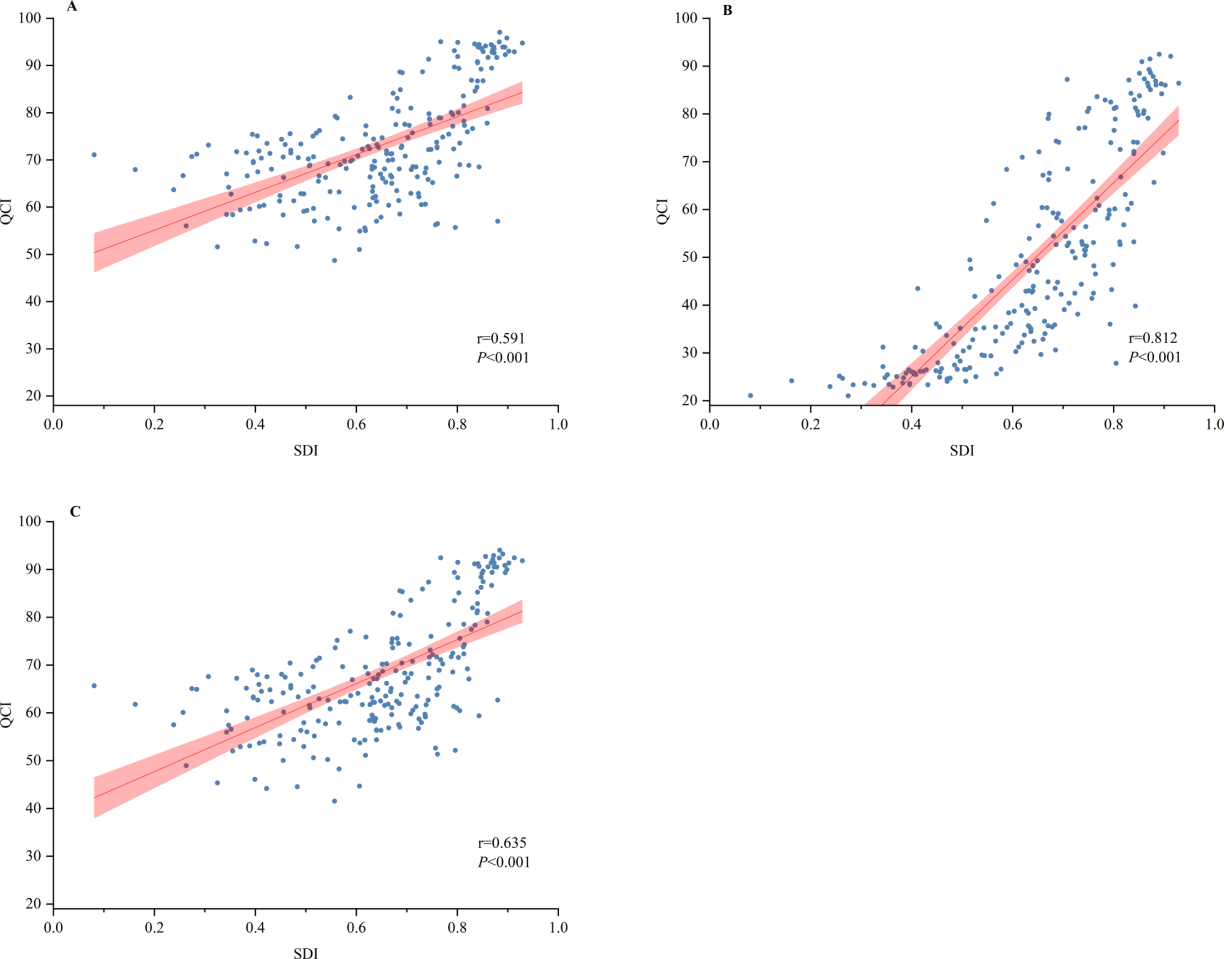
Association between country-level sociodemographic index (SDI) and quality of care index (QCI) for leukaemia, brain and central nervous system (CNS) tumours and both two tumours in 2019. (A) The association between SDI regions and QCI for leukaemia. (B) The association between SDI regions and QCI for brain and CNS tumours. (C) The association between SDI regions and QCI for both two childhood cancers. The correlation coefficient and p values were calculated to show the association between country-level SDI with the QCI for leukaemia and CNS tumours.

The QCI was closely correlated with the sociodemographic level for both leukaemia and the brain and CNS tumours. Online supplemental figure 2 shows the trend of the QCI in different SDI regions from 1990 to 2019. In high, high-middle and middle SDI countries, the QCI for leukaemia was above the global average, while in low-middle and low SDI countries, it was below the global average. For brain and CNS tumours, the disparity between SDI levels was pronounced, with QCI in low SDI countries being only 35.74% of that in high SDI countries. As shown in figure 3, the country-level QCI of leukaemia was correlated with the country-level SDI (r=0.591, p<0.001), and a similar trend was observed for CNS tumour (r=0.812, p<0.001).

From 1990 to 2019, countries in high, high-middle and low SDI levels have experienced an increasing trend, with the EAPC of 0.32 (95% CI: 0.29 to 0.35), 0.27 (95% CI: 0.24 to 0.30), 0.16 (95% CI: 0.13 to 0.18), respectively. However, there was a continuous downward trend in the middle SDI and low-middle SDI regions, with EAPC of −0.13 (95% CI: −0.16 to −0.09) and −0.19 (95% CI: −0.20 to −0.18), respectively. This phenomenon suggests that the differences in the quality of leukaemia care between middle and low-middle SDI regions may expand with other regions in the future, triggering further attention to middle SDI and low-middle SDI regions. From 1990 to 2019, the QCI of brain and CNS tumours showed an overall increasing trend (table 1).

**Table 1:** Estimated average percentage change (EAPC) and 95% CI of leukaemia and brain and central nervous system (CNS) tumours in 1990 and 2019, by sociodemographic index (SDI) regions and by genders

	Leukaemia	Brain and central nervous system tumours	Two childhood cancers
Both			
Global	−0.05 (−0.07, –0.03)	1.45 (1.41, 1.50)	−0.02 (−0.04, 0.01)
High SDI	0.32 (0.29, 0.35)	0.56 (0.52, 0.60)	0.35 (0.32, 0.38)
High-middle SDI	0.27 (0.24, 0.30)	2.13 (2.01, 2.25)	0.36 (0.33, 0.39)
Middle SDI	−0.13 (−0.16, –0.09)	2.09 (2.04, 2.14)	−0.07 (−0.12, –0.03)
Low-middle SDI	−0.19 (−0.20, –0.18)	1.45 (1.36, 1.54)	−0.26 (−0.28, –0.25)
Low SDI	0.16 (0.13, 0.18)	0.88 (0.82, 0.95)	0.07 (0.04, 0.10)
Girl			
Global	−0.03 (−0.06, –0.01)	1.46 (1.41, 1.51)	−0.02 (−0.04, 0.01)
High SDI	0.23 (0.21, 0.26)	0.52 (0.49, 0.56)	0.27 (0.25, 0.29)
High-middle SDI	0.22 (0.18, 0.26)	2.06 (1.95, 2.18)	0.31 (0.27, 0.35)
Middle SDI	−0.13 (–0.17, –0.10)	2.10 (2.04, 2.16)	−0.09 (–0.13, –0.05)
Low-middle SDI	−0.15 (–0.16, –0.15)	1.53 (1.43, 1.63)	−0.27 (–0.28, –0.26)
Low SDI	0.13 (0.11, 0.15)	0.83 (0.75, 0.90)	0.02 (–0.01, 0.04)
Boy			
Global	−0.03 (–0.05, –0.01)	1.43 (1.39, 1.47)	0.03 (0.00, 0.05)
High SDI	0.41 (0.37, 0.46)	0.59 (0.55, 0.64)	0.43 (0.39, 0.47)
High-middle SDI	0.34 (0.31, 0.36)	2.17 (2.05, 2.30)	0.43 (0.41, 0.46)
Middle SDI	−0.09 (–0.12, –0.05)	2.03 (1.99, 2.08)	−0.03 (–0.07, 0.01)
Low-middle SDI	−0.17 (–0.18, –0.16)	1.39 (1.30, 1.47)	−0.18 (–0.20, –0.16)
Low SDI	0.33 (0.29, 0.37)	0.92 (0.85, 0.98)	0.29 (0.25, 0.32)

### Gender and age trend of QCI

**Figure 4:**
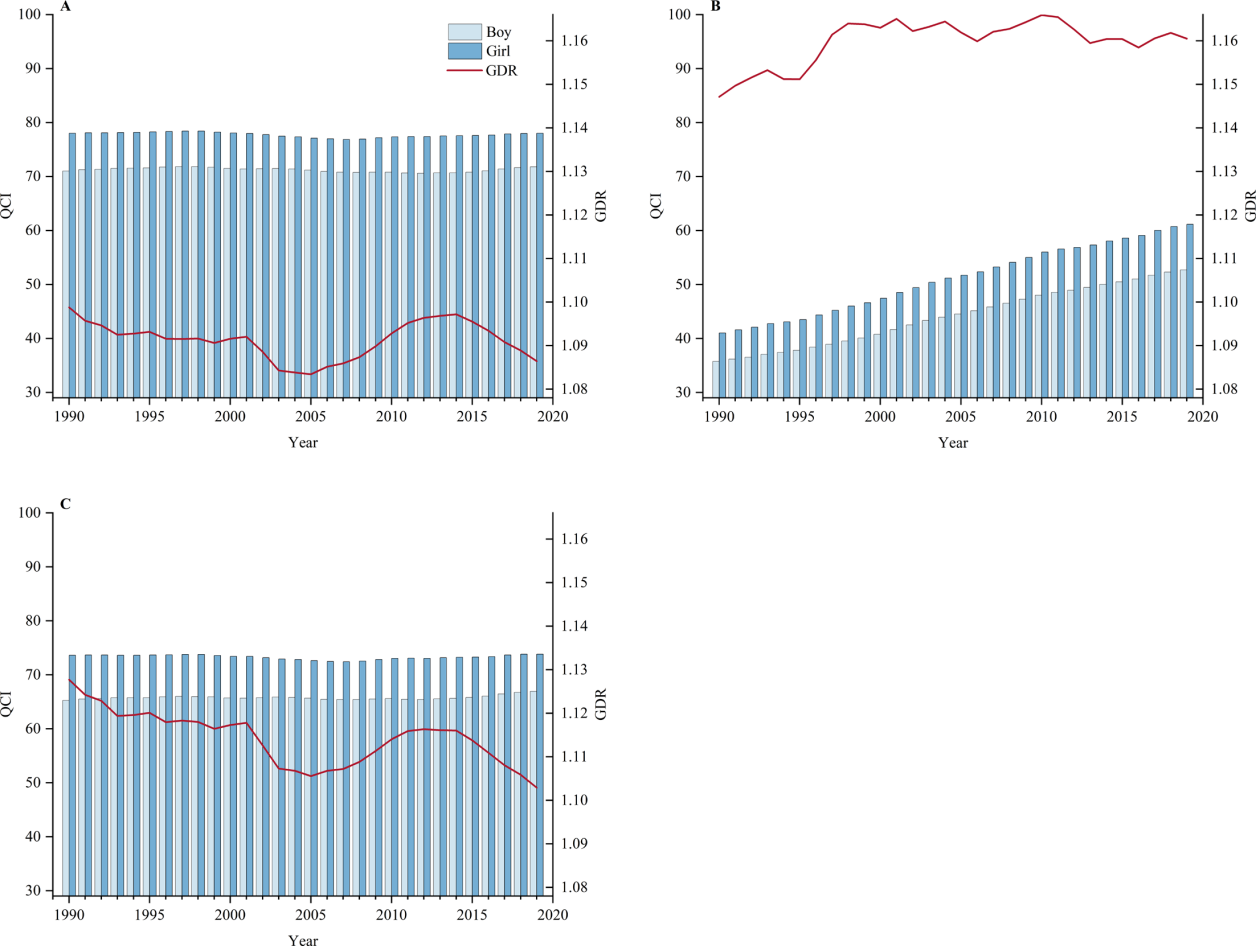
The gender disparity ratio (GDR) and quality of care index (QCI) for leukaemia and brain and central nervous system (CNS) tumours, by genders, from 1990 to 2019. (A) The QCI for leukaemia in boys and girls, and GDR at global level, from 1990 to 2019. (B) The QCI for CNS tumours in boys and girls, and GDR at global level, from 1990 to 2019. (C) The QCI for both two tumours in boys and girls, and GDR at global level, from 1990 to 2019. GDR was calculated as the ratio of QCI in girls divided by the QCI score in boys, where GDR >1 represented a better QCI level in girls than boys.

The QCI of leukaemia and CNS tumours across genders was explored through the calculated GDR. Overall, the QCI of boys was lower than that of girls, for both leukaemia and the CNS tumour. The gender difference of brain and CNS tumour fluctuated, and the GDR increased from 1.147 in 1990 to 1.160 in 2019, suggesting that the gender difference was gradually expanding. From 1990 to 2019, QCI for leukaemia was of a significantly decreasing trend, with EAPC of −0.03 (95% CI: −0.05 to −0.01) and −0.03 (95% CI: −0.06 to −0.01) for boys and girls separately (figure 4 and online supplemental table 3).

The QCI of leukaemia showed a decreasing trend with age, with the lowest QCI of 64.15 in the 15–19 years age group. The QCI of CNS tumour fluctuated with age, that lower QCIs were observed in age groups less than 1 year and 5–9 years of age, while a high QCI was observed in 15–19 years of age (online supplemental table 3 and online supplemental figure 3).

## Discussion

Childhood cancer is a global public health issue, and the WHO has proposed a Global Initiative with the goal of increasing the survival rate of children with cancer globally to at least 60% by 2030 while reducing their suffering and improving their quality of life. In this study, we established a QCI to represent the quality of childhood cancer care, and the results suggested that overall QCI was associated with sociodemographic levels, while the QCI of leukaemia in the middle SDI and low-middle SDI regions showed a decreasing temporal trend, and the gender disparity of QCI for CNS tumours increased over 30 years.

In this study, we focused on two kinds of childhood cancer types, that is, leukaemia and CNS tumour, and quality of cancer care significantly valued in the two. Leukaemia was of the highest burden out of all childhood cancers,^13^ which has caused 43 193 deaths and 3 544 099.33 DALYs in 2019, taking a proportion of 32.67% and 32.96% out of the total childhood cancer occurring in 0–19 years of age. Leukaemia may appear at all ages, but different subtypes of leukaemia have different prevalence rates at different ages.^14^ Acute lymphoblastic leukaemia (ALL) is most common in early childhood and is more prevalent in males than in females.^15^ Acute myeloid leukaemia (AML) is highly prevalent in the elderly population, whereas chronic myeloid leukaemia (CML) and chronic lymphoid leukaemia (CLL) are rare in young children. Many genetic factors have been shown to be associated with an increased risk of ALL, including Down syndrome, germline mutations in PAX5 and ETV6 and polymorphic variants in specific genes.^16^ Prospective cohort studies based on older adults have found that an increase in leukaemia may also be associated with complications of haematologic malignancies, and increased exposure to radiotherapy and chemotherapy,^17^ but the extent of their impact on the development of leukaemia in children is unclear. Significantly higher 5-year relative survival rates for ALL and AML in adolescents compared with older patients, reflecting inequalities in access to care between children and older patients, differences in treatment regimens, and more aggressive disease in older leukaemia patients.^18^ With unclear carcinogenesis of leukaemia disease, early detection of specific symptoms, such as hepatomegaly, splenomegaly, bruising, fever, limb and bone pain, pallor, fatigue and anorexia, and followed by appropriate treatment and high-quality care, is the primary strategy for leukaemia prevention and control. CNS tumours are the second most common childhood malignancy and the most common solid tumour in children, and are the most common cause of death among all childhood cancers. Given the unclear process of CNS tumour development, enhancing the quality of childhood cancer care is the key to improving childhood cancer prognosis as well as to reducing the disease burden, as recorded that regimens for management have been emphasised as key pillars in the WHO global initiative. A recent systematic review by Uwishema *et al*^19^ highlights the significant burden of CNS tumours in Africa, where limited access to diagnostic and treatment facilities exacerbates poor outcomes. While their study focuses on the African context, it underscores the global disparities in CNS tumour care and the urgent need for targeted interventions in low-resource settings to improve outcomes for children and adolescents worldwide.^19^

In this study, we observed a correlation between the quality of leukaemia and CNS tumour care and social development levels. This observation was similarly reported in the global estimation that high-income regions had higher QCIs than the global average values,^9^ which could be explained by the country’s capacity to deliver qualified childhood cancer services. The NCD Country Capacity Survey conducted by the WHO showed that over 90% of HICs had the ability to deliver fundamental cancer diagnosis and treatment services including pathology services (laboratories), cancer surgery, chemotherapy and radiotherapy. However, 55% of LMICs reported that none of these services were available.^20^ This greatly varied service capacity would explain the disparity of childhood cancer QCI across countries in different social development levels. Second, financial expenses were also a barrier against a high QCI, especially in LMICs. Lack of universal health coverage leads to significant inequalities in access to and quality of information on cancer in LMICs, and the capacity for early diagnosis and management of paediatric cancer cases remains limited and often lacks effective investment in childhood cancer patients. In contrast, universal health coverage efforts in HICs have resulted in greater access to early diagnosis and treatment and quality services for more childhood cancer patients.^21 22^ Meanwhile, factors from the parents’ perspective would also be a barrier against timely, high-quality childhood cancer care.^23^ The most common reasons for treatment abandonment include poverty, a lack of interest in their own disease, cultural myths, feelings of guilt and/or social discrimination among their peers.

Some studies have shown that the incidence of and deaths from leukaemia have increased globally over the past three decades, with higher incidence and lower mortality rates in regions with higher economic levels, reflecting years of relentless efforts at the prevention, early detection, diagnosis and treatment of haematologic malignancies.^24^ This study observed an increasing QCI since 1990, while the QCI of leukaemia in the low SDI and low-middle SDI levels significantly decreased. The decreasing QCI trend may be related to the highly increased disease burden of leukaemia in low SDI and low-middle SDI levels and an unproportionate increasing ability to deliver corresponding leukaemia healthcare services in these countries. GBD reported that 37.43% of the incident cases were emerging in low and low-middle countries in 2019, which was 1.31 times higher than the value of 28.53% in 1990. However, the improvement of national ability for leukaemia early detection, treatment and long-term care was limited. For example, in Asia, 5-year survival estimates for LMICs range from 34.3% to 73.1%, compared with 77.1% to 85.0% in HICs.^25 26^ Early deaths due to infection, haemorrhage and abandonment of treatment are more frequent, with up to 50%–60% of children abandoning treatment in some areas.^27^ Meanwhile, children with leukaemia needed long-term care, during which a family approach was also valued. However, the related social intervention and support were far from satisfactory in low SDI and low-middle SDI countries. Lutz Goldbeck certified that promoting communication between parents about their coping strategies and about the reactions of their child could improve the goodness of fit of the family’s joint efforts in coping with childhood cancer.^28^ However, delayed diagnosis, early deaths, abandonment of treatment and increased relapse rates are major challenges for families of leukaemia children patients in low-income countries and disparities in the capacity of health services contribute to the lack of timely access to effective health resources for local children.^26 29^ Thus, the inconsistency in fasting increases leukaemia burden with limited healthcare service ability and calls for more attention to be paid in low SDI and low-middle SDI regions with a high incidence of leukaemia, especially in the development of a resilient and sustainable health system to deliver timely, affordable and high-quality childhood cancer care to respond to the emerging childhood cancer burden. It also requires us to raise health awareness, increase investment in healthcare, strengthen global partnerships to improve imbalances in socioeconomic development and reduce the burden of disease in LMICs.

The QCI of childhood cancer differed between the sexes. Two observations were identified in this study. First, the QCI for childhood cancer was higher in girls than that in boys. This may be related to the high burden and worse prognosis of childhood cancer in boys than in girls.^10^ Sex genotype plays a significant role in gender disparity in childhood cancer care. A study by Soon *et al* showed that all tumours had a higher incidence in boys, regardless of tumour subtype, patient age or region.^30 31^ It has also been confirmed that the incidence and mortality rates of different subtypes of leukaemia in boys tend to be higher than those in girls in different SDI regions. The incidence of different subtypes of leukaemia is similar in all countries, with the largest gender differences in AML and CLL, and smaller gender differences in ALL, which is generally male-dominated.^32^ We also need to admit that this observation could be biased if a higher proportion of girls remained undiagnosed of childhood cancer due to boys’ preferential attention in certain cultural backgrounds. To address this issue, it is crucial for policymakers to consider gender as a factor in the design of childhood cancer care programmes, particularly in LMICs, where disparities in care access are often exacerbated by socio-economic and cultural factors. Promoting gender-sensitive healthcare interventions and ensuring equal access to diagnosis, treatment and supportive care for both sexes should be prioritised in national cancer strategies. Additionally, further research is needed to explore the underlying causes of these disparities and develop targeted interventions. Second, the gender disparity of QCI in brain and CNS tumour showed an enlarging temporal trend. Distinguished subtype distribution in girls and boys could explain the increasing gender disparity in QCI. There are more than 100 different histological subtypes of CNS tumours, and the incidence varies according to age and histological subtype.^33 34^ The WHO’s CNS tumours classification released in 2021 uses extensive data from molecular testing, confirming higher mortality rates for some subtypes.^35^ Some studies have confirmed the high incidence of malignant CNS tumours in boys and the high incidence of non-malignant tumours in girls, both of which are on an upward trend,^36^ and brain and CNS tumours are the most common causes of cancer death in boys.^36 37^ Although the quality of care for girls with CNS tumours is higher than that of boys globally, the GDR value of <1 in the low SDI region suggests that boys receive a higher quality of care than females in this region, whereas the GDR value of close to 1 in the high SDI region suggests that the quality of care received by boys and girls is almost equal, which is in line with the findings of our study (online supplemental table 3).^9^ However, potential confounders, such as differences in healthcare access, socioeconomic status and regional healthcare policies, may influence these results and should be considered when interpreting the gender disparities in QCI. Other possible explanations should be further explored. Nevertheless, the alarmingly low QCI for brain and CNS tumour in boys should be paid more attention to and deserve further innovation research.

According to previous studies, there are large differences in the quality of care between adult and childhood patients with cancer. Globally, the QCI of paediatric leukaemia patients was lower than that of adult leukaemia patients, and the quality of care of paediatric leukaemia patients in low SDI and low-middle SDI regions was rather higher than that of adult leukaemia patients.^10^ In addition, a previous study also found that the patients in their early adulthood with CNS tumours have higher QCI. Paediatric patients had better quality of care in the high SDI and high-middle SDI regions, while the QCI for CNS tumours was poor for all ages in the low and low-middle SDI regions, which was consistent with our study. These differences may be due to the different SDI levels and the subtypes of tumours.^9^

Results of this study will draw public attention to childhood cancer patients and the quality of care, especially focusing on the unmet needs in LMICs and the poorer regions of HICs, with a view to formulating better policies and regulations to enable them to receive better care.

This study has several strengths and advantages. We systematically estimated the quality of care for two common types of childhood cancer at global, regional and national levels. The results of this study could provide essential data on vulnerable populations to better implement the *CureAll* approach to accomplish the global initiative by increasing access, advancing quality and saving lives. The study also has some limitations. First, our results may be interpreted with caution in some areas due to the limitations of the IHME-GBD dataset in national data registries. These limitations include potential reporting biases, incomplete data and varying standards of data collection across different countries. Second, we did not assess variables such as disease subtypes and ethnicity separately due to data unavailability. Additionally, the completeness of childhood cancer registration may vary across countries, particularly those with differing socioeconomic status. In countries with well-established cancer registries, data is generally more complete, while in low-income or less-developed countries, challenges such as underreporting and limited resources may affect data accuracy and availability. Furthermore, the cross-sectional nature of this study prevents us from drawing causal conclusions about the relationship between quality of care and the various factors considered. These discrepancies and methodological constraints should be considered when interpreting the findings from the GBD dataset.

## Conclusions

In summary, we estimated the quality of care for children with leukaemia and CNS tumours. Overall, QCI showed an improving temporal trend, and QCI was positively associated with country-level social development levels, while the QCI of leukaemia in the middle and low-middle SDI regions showed a decreasing trend. Boys had a lower QCI level than girls, and the sex disparity was increasing in CNS tumours. This estimation highlighted the vulnerable regions and populations in accessing high-quality childhood cancer care. Countries with low social development levels and boys should be prioritised in policy interventions to reduce health disparities. To address these issues, policymakers in LMICs should focus on improving access to quality care, particularly for boys and populations in lower SDI regions. Ensuring equitable healthcare policies, increasing access to early diagnosis and enhancing treatment options will be crucial in addressing these disparities. Efforts to implement gender-sensitive approaches and targeted interventions can help bridge the care gaps and ultimately improve survival rates.

## supplementary material

10.1136/bmjopen-2024-093397 online supplemental file 1

## Data Availability

The datasets generated and/or analysed during the current study are available in the Global Health Data Exchange (http://ghdx.healthdata.org).

## References

[1] Steliarova-Foucher E, Colombet M, Ries LAG (2017). International incidence of childhood cancer, 2001-10: a population-based registry study. Lancet Oncol.

[2] Organization GWH (2021). WHO global initiative for childhood cancer. Increasing access, advancing quality, saving lives.

[3] Atun R, Bhakta N, Denburg A (2020). Sustainable care for children with cancer: a Lancet Oncology Commission. Lancet Oncol.

[4] Organization WH (2020). WHO global initiative for childhood cancer: an overview.

[5] Rodriguez-Galindo C, Friedrich P, Alcasabas P (2015). Toward the Cure of All Children With Cancer Through Collaborative Efforts: Pediatric Oncology As a Global Challenge. J Clin Oncol.

[6] Azadnajafabad S, Saeedi Moghaddam S, Keykhaei M (2023). Expansion of the quality of care index on breast cancer and its risk factors using the global burden of disease study 2019. Cancer Med.

[7] Aryannejad A, Tabary M, Ebrahimi N (2021). Global, regional, and national survey on the burden and quality of care of pancreatic cancer: a systematic analysis for the Global Burden of Disease study 1990-2017. Pancreatology.

[8] Hanifiha M, Ghanbari A, Keykhaei M (2022). Global, regional, and national burden and quality of care index in children and adolescents: A systematic analysis for the global burden of disease study 1990-2017. PLoS ONE.

[9] Mohammadi E, Ghasemi E, Azadnajafabad S (2021). A global, regional, and national survey on burden and Quality of Care Index (QCI) of brain and other central nervous system cancers; global burden of disease systematic analysis 1990-2017. PLoS One.

[10] Keykhaei M, Masinaei M, Mohammadi E (2021). A global, regional, and national survey on burden and Quality of Care Index (QCI) of hematologic malignancies; global burden of disease systematic analysis 1990-2017. Exp Hematol Oncol.

[11] GBD 2019 Demographics Collaborators (2020). Global age-sex-specific fertility, mortality, healthy life expectancy (HALE), and population estimates in 204 countries and territories, 1950–2019: a comprehensive demographic analysis for the Global Burden of Disease Study 2019. Lancet.

[12] GBD 2019 Adolescent Mortality Collaborators (2021). Global, regional, and national mortality among young people aged 10-24 years, 1950-2019: a systematic analysis for the Global Burden of Disease Study 2019. Lancet.

[13] Johnston WT, Lightfoot TJ, Simpson J (2010). Childhood cancer survival: a report from the United Kingdom Childhood Cancer Study. Cancer Epidemiol.

[14] Juliusson G, Lazarevic V, Hörstedt A-S (2012). Acute myeloid leukemia in the real world: why population-based registries are needed. Blood.

[15] Lim JY-S, Bhatia S, Robison LL (2014). Genomics of racial and ethnic disparities in childhood acute lymphoblastic leukemia. Cancer.

[16] Hunger SP, Mullighan CG (2015). Acute Lymphoblastic Leukemia in Children. N Engl J Med.

[17] Hulegårdh E, Nilsson C, Lazarevic V (2015). Characterization and prognostic features of secondary acute myeloid leukemia in a population-based setting: a report from the Swedish Acute Leukemia Registry. Am J Hematol.

[18] Juliusson G, Hough R, Stark DP, Vassal G (2016). Tumors in adolescents and young adults.

[19] Uwishema O, Frederiksen KS, Badri R (2023). Epidemiology and etiology of brain cancer in Africa: A systematic review. Brain Behav.

[20] Organization WH (2021). CureAll framework: WHO global initiative for childhood cancer: increasing access, advancing quality, saving lives.

[21] Fung A, Horton S, Zabih V (2019). Cost and cost-effectiveness of childhood cancer treatment in low-income and middle-income countries: a systematic review. BMJ Glob Health.

[22] Hayati H, Kebriaeezadeh A, Ehsani MA (2016). Treatment costs for pediatrics acute lymphoblastic leukemia; comparing clinical expenditures in developed and developing countries: A review article. Int J Pediatr.

[23] Ni X, Li Z, Li X (2022). Socioeconomic inequalities in cancer incidence and access to health services among children and adolescents in China: a cross-sectional study. Lancet.

[24] Zhang N, Wu J, Wang Q (2023). Global burden of hematologic malignancies and evolution patterns over the past 30 years. Blood Cancer J.

[25] Nakata K, Okawa S, Fuji S (2021). Trends in survival of leukemia among children, adolescents, and young adults: A population-based study in Osaka, Japan. Cancer Sci.

[26] Pui C-H, Yang JJ, Bhakta N (2018). Global efforts toward the cure of childhood acute lymphoblastic leukaemia. Lancet Child Adolesc Health.

[27] Friedrich P, Lam CG, Itriago E (2015). Magnitude of Treatment Abandonment in Childhood Cancer. PLoS One.

[28] Goldbeck L (2001). Parental coping with the diagnosis of childhood cancer: gender effects, dissimilarity within couples, and quality of life. Psychooncology.

[29] Antillón FG, Blanco JG, Valverde PD (2017). The treatment of childhood acute lymphoblastic leukemia in Guatemala: Biologic features, treatment hurdles, and results. Cancer.

[30] Soon WC, Goacher E, Solanki S (2021). The role of sex genotype in paediatric CNS tumour incidence and survival. Childs Nerv Syst.

[31] Williams LA, Richardson M, Marcotte EL (2019). Sex ratio among childhood cancers by single year of age. Pediatr Blood Cancer.

[32] Miranda-Filho A, Piñeros M, Ferlay J (2018). Epidemiological patterns of leukaemia in 184 countries: a population-based study. Lancet Haematol.

[33] Johnson KJ, Cullen J, Barnholtz-Sloan JS (2014). Childhood brain tumor epidemiology: a brain tumor epidemiology consortium review. Cancer Epidemiol Biomarkers Prev.

[34] Ward E, DeSantis C, Robbins A (2014). Childhood and adolescent cancer statistics, 2014. CA Cancer J Clin.

[35] Smith HL, Wadhwani N, Horbinski C (2022). Major Features of the 2021 WHO Classification of CNS Tumors. Neurotherapeutics.

[36] Miller KD, Ostrom QT, Kruchko C (2021). Brain and other central nervous system tumor statistics, 2021. CA Cancer J Clin.

[37] Miller KD, Fidler-Benaoudia M, Keegan TH (2020). Cancer statistics for adolescents and young adults, 2020. CA Cancer J Clin.

