# Supplementary material for: Global, regional and national burden and quality of care index (QCI) of leukaemia and brain and central nervous system tumours in children and adolescents aged 0–19 years: a systematic analysis of the Global Burden of Disease Study 1990–2019
Source: BMJ Open. 2025 Mar 22;15(3):e093397. doi: 10.1136/bmjopen-2024-093397 (PMC11931942; doi:10.1136/bmjopen-2024-093397)
Supplement: online supplemental file 1 [file bmjopen-15-3-s001.docx]

**Supplement.**

Table 1：Death number and rate of <20 years old childhood cancer patients in 1990 and 2019, by sociodemographic index (SDI) regions.

|  | Leukemia | | | | Brain and central nervous system cancer | | | |
| --- | --- | --- | --- | --- | --- | --- | --- | --- |
|  | 1990number | 2019number | 1990rate | 2019rate | 1990number | 2019number | 1990rate | 2019rate |
| **Death** |  |  |  |  |  |  |  |  |
| Global | 82302 (107758, 62309) | 43193 (49284, 37486) | 3.62 (4.74, 2.74) | 1.67 (1.91, 1.45) | 29735 (47585, 19859) | 23538 (27496, 18051) | 1.31 (2.09, 0.87) | 0.91 (1.07, 0.70) |
| High SDI | 3932 (4061, 3761) | 1647 (1763, 1541) | 1.68(1.73, 1.61) | 0.75 (0.80, 0.70) | 2272 (2936, 2022) | 1677 (1869, 1200) | 0.97 (1.25, 0.86) | 0.76 (0.85, 0.54) |
| High-middle SDI | 15760 (17879, 13452) | 5104 (5771, 4360) | 3.89 (4.41, 3.32) | 1.56 (1.76, 1.33) | 6171 (8060, 4804) | 3380 (3873, 2386) | 1.52 (1.99, 1.19) | 1.03 (1.18, 0.73) |
| Middle SDI | 31635 (38555, 25059) | 13427 (15307, 11629) | 4.12 (5.03, 3.27) | 1.82 (2.08, 1.58) | 10670 (16849, 7094 ) | 6813 (8057, 5006) | 1.39 (2.20, 0.93) | 0.92 (1.09, 0.68) |
| Low-middle SDI | 18873 (27437, 11631) | 10711 (12822, 8970) | 3.31 (4.81, 2.04) | 1.54 (1.84, 1.29) | 7384 (13986, 4158) | 6459 (7865, 5165) | 1.29 (2.45, 0.73) | 0.93 (1.13, 0.74) |
| Low SDI | 12055 (21852, 5695) | 12265 (15853, 9336) | 4.08 (7.40, 1.93) | 2.05 (2.66, 1.56) | 3226 (6671, 1559) | 5194 (6861, 3824) | 1.09 (2.26, 0.53) | 0.87 (1.15, 0.64) |
| **DALY** |  |  |  |  |  |  |  |  |
| Global | 6859937 (9042373, 5136004) | 3544099 (4061709, 3056968) | 301.73 (397.72, 225.90) | 137.41 (157.47, 118.52) | 2452771 (3947887, 1619067) | 1923038 (2248519, 1474554) | 107.88 (173.64, 71.21) | 74.56 (87.18, 57.17) |
| High SDI | 317965 (329137, 304513) | 134888 (144817, 125582) | 135.79 (140.56, 130.04) | 61.08 (65.58, 56.87) | 184120 (238719, 163864) | 135949 (151553, 97023) | 78.63 (101.95, 69.98) | 61.56 (68.63, 43.94) |
| High-middle SDI | 1300258 (1484086, 1104168) | 417368 (473903, 356190) | 320.85 (366.21, 272.47) | 127.26 (144.50, 108.61) | 503574 (661039, 389567) | 274751 (314839, 194405) | 124.26 (163.12, 96.13) | 83.77 (96.00, 59.28) |
| Middle SDI | 2627076 (3226625, 2071536) | 1085963 (1238670, 938803) | 342.55 (420.72, 270.11) | 147.44 (168.17, 127.46) | 875910 (1386094, 575783) | 551953 (653575, 405936) | 114.21 (180.73, 75.08) | 74.94 (88.73, 55.11) |
| Low-middle SDI | 1577763 (2317215, 961824) | 870409 (1048125, 727878) | 276.51 (406.11, 168.57) | 125.17 (150.73, 104.68) | 616367 (1176109, 341911) | 528276 (643830, 421153) | 108.02 (206.12, 59.92) | 75.97 (92.59, 60.57) |
| Low SDI | 1032926 (1882543, 481293) | 1032245 (1340058, 775513) | 349.70 (637.35, 162.95) | 172.92 (224.48, 129.91) | 271888 (565607, 129452) | 430859 (573925, 316095) | 92.05 (191.49, 43.83) | 72.18 (96.14, 52.95) |

Table 2：

Quality of care index of Leukemia, Brain and central nervous system cancer, and ther sum in 1990 and 2019, by Global Burden of Disease (GBD) regions.

| Location | Leukemia | | | Brain and central nervous system cancer | | | Both | | |
| --- | --- | --- | --- | --- | --- | --- | --- | --- | --- |
|  | 1990QCI | 2019QCI | EAPC (95% CI) | 1990QCI | 2019QCI | EAPC (95% CI) | 1990QCI | 2019QCI | EAPC (95% CI) |
| **Global** | 74.68 | 74.71 | -0.05 (-0.07, -0.03) | 38.02 | 56.59 | 1.44 (1.40, 1.49) | 69.51 | 70.16 | -0.02 (-0.04, 0.01) |
| **Southeast Asia, East Asia, and Oceania** | 80.71 | 84.10 | 0.08 (0.05, 0.11) | 33.34 | 67.59 | 2.66 (2.55, 2.77) | 76.04 | 80.82 | 0.16 (0.13, 0.19) |
| **East Asia** | 82.03 | 88.51 | 0.21 (0.18, 0.24) | 34.48 | 74.07 | 2.93 (2.78, 3.08) | 77.19 | 85.37 | 0.31 (0.28, 0.35) |
| China | 81.86 | 88.64 | 0.22 (0.20, 0.25) | 33.78 | 74.32 | 3.02 (2.87, 3.18) | 76.99 | 85.52 | 0.33 (0.30, 0.36) |
| Democratic People's Republic of Korea | 87.10 | 79.26 | -0.35 (-0.38, -0.31) | 42.01 | 43.02 | 0.17 (-0.10, 0.44) | 83.61 | 73.60 | -0.46 (-0.51, -0.42) |
| Taiwan (Province of China) | 86.80 | 89.46 | 0.05 (0.03, 0.07) | 66.00 | 79.11 | 0.67 (0.64, 0.71) | 82.62 | 86.68 | 0.14 (0.12, 0.15) |
| **Southeast Asia** | 75.32 | 72.65 | -0.12 (-0.13, -0.12) | 27.57 | 39.29 | 1.23 (1.18, 1.28) | 71.26 | 67.98 | -0.16 (-0.17, -0.16) |
| Cambodia | 75.98 | 75.55 | 0.00 (-0.02, 0.02) | 23.24 | 33.65 | 1.38 (1.28, 1.49) | 72.34 | 70.40 | -0.07 (-0.10, -0.05) |
| Indonesia | 75.77 | 71.34 | -0.2 (-0.22, -0.18) | 24.92 | 32.83 | 0.86 (0.79, 0.93) | 71.74 | 66.17 | -0.28 (-0.29, -0.26) |
| Lao People's Democratic Republic | 74.59 | 73.4 | -0.08 (-0.08, -0.07) | 21.80 | 29.25 | 0.96 (0.86, 1.06) | 70.58 | 68.06 | -0.14 (-0.15, -0.13) |
| Malaysia | 62.91 | 60.64 | -0.19 (-0.26, -0.13) | 34.55 | 53.30 | 1.59 (1.54, 1.64) | 59.71 | 59.16 | -0.09 (-0.16, -0.02) |
| Maldives | 75.79 | 78.9 | 0.14 (0.07, 0.22) | 27.92 | 61.24 | 2.98 (2.84, 3.11) | 70.94 | 75.16 | 0.23 (0.13, 0.32) |
| Myanmar | 76.21 | 75.73 | -0.01 (-0.01, 0.00) | 23.70 | 32.75 | 1.07 (0.91, 1.22) | 72.55 | 70.97 | -0.06 (-0.07, -0.06) |
| Philippines | 75.55 | 72.87 | -0.15 (-0.16, -0.13) | 28.00 | 33.75 | 0.53 (0.44, 0.62) | 71.20 | 68.19 | -0.15 (-0.17, -0.14) |
| Sri Lanka | 70.50 | 72.97 | 0.15 (0.09, 0.22) | 36.54 | 59.17 | 1.70 (1.56, 1.83) | 67.92 | 70.42 | 0.12 (0.06, 0.19) |
| Thailand | 83.81 | 84.87 | 0.05 (0.02, 0.09) | 35.71 | 58.26 | 1.84 (1.77, 1.92) | 78.34 | 80.35 | 0.10 (0.07, 0.14) |
| Timor-Leste | 77.74 | 75.04 | -0.11 (-0.13, -0.09) | 23.11 | 31.18 | 1.15 (1.00, 1.31) | 74.23 | 69.65 | -0.21 (-0.23, -0.20) |
| Viet Nam | 54.41 | 55.59 | 0.10 (0.03, 0.18) | 31.22 | 50.33 | 1.76 (1.69, 1.84) | 51.55 | 54.33 | 0.21 (0.12, 0.30) |
| **Oceania** | 72.55 | 74.37 | 0.10 (0.08, 0.08) | 27.32 | 27.90 | -0.03 (-0.11, 0.05) | 67.09 | 68.09 | 0.05 (0.03, 0.07) |
| Fiji | 59.74 | 60.40 | -0.02 (-0.07, 0.03) | 32.32 | 34.03 | 0.16 (0.08, 0.24) | 56.23 | 56.85 | -0.03 (-0.08, 0.02) |
| Kiribati | 76.84 | 76.21 | -0.03 (-0.04, -0.02) | 23.87 | 25.02 | 0.17 (0.13, 0.20) | 72.97 | 71.45 | -0.08 (-0.09, -0.07) |
| Marshall Islands | 70.28 | 69.21 | 0.02 (-0.02, 0.06) | 27.50 | 29.42 | 0.25 (0.15, 0.35) | 64.11 | 62.67 | -0.02 (-0.06, 0.01) |
| Micronesia (Federated States of) | 69.75 | 68.22 | -0.07 (-0.09, -0.05) | 27.01 | 34.07 | 0.80 (0.74, 0.85) | 64.11 | 62.31 | -0.10 (-0.12, -0.08) |
| Papua New Guinea | 74.49 | 75.44 | 0.04 (0.03, 0.05) | 25.29 | 26.45 | 0.06 (-0.01, 0.14) | 68.77 | 68.97 | 0.00 (-0.02, 0.02) |
| Samoa | 71.59 | 73.26 | -0.01 (-0.04, 0.02) | 35.09 | 43.95 | 0.22 (0.06, 0.38) | 65.53 | 67.11 | -0.05 (-0.10, -0.01) |
| Solomon Islands | 71.68 | 71.90 | 0.01 (-0.01, 0.03) | 27.98 | 31.15 | 0.23 (0.13, 0.34) | 66.35 | 65.98 | -0.03 (-0.05, -0.01) |
| Tonga | 70.88 | 69.76 | -0.04 (-0.06, -0.03) | 30.66 | 34.45 | 0.40 (0.34, 0.47) | 63.43 | 62.20 | -0.07 (-0.09, -0.04) |
| Vanuatu | 70.80 | 70.46 | 0.01 (-0.01, 0.03) | 26.61 | 27.45 | 0.02 (-0.06, 0.10) | 64.18 | 63.33 | -0.03 (-0.05, 0.00) |
| **Central Europe, Eastern Europe, and Central Asia** | 67.79 | 72.75 | 0.32 (0.26, 0.37) | 31.45 | 42.50 | 1.13 (1.03, 1.23) | 61.31 | 63.78 | 0.18 (0.13, 0.24) |
| **Central Asia** | 75.15 | 69.91 | -0.27 (-0.30, -0.25) | 28.73 | 36.61 | 0.97 (0.86, 1.09) | 70.70 | 61.83 | -0.54 (-0.59, -0.49) |
| Armenia | 72.92 | 72.64 | 0.22 (0.09, 0.36) | 30.53 | 44.76 | 1.50 (1.42, 1.58) | 64.32 | 63.79 | 0.14 (0.04, 0.25) |
| Azerbaijan | 80.69 | 80.34 | 0.04 (0.00, 0.07) | 27.39 | 35.87 | 1.10 (0.89, 1.30) | 75.80 | 74.53 | -0.01 (-0.05, 0.03) |
| Georgia | 80.05 | 74.72 | -0.25 (-0.36, -0.14) | 35.29 | 39.03 | 0.15 (0.06, 0.24) | 76.98 | 67.25 | -0.51 (-0.64, -0.37) |
| Kazakhstan | 65.93 | 62.94 | -0.03 (-0.18, 0.12) | 30.75 | 42.50 | 1.45 (1.23, 1.66) | 63.20 | 56.76 | -0.29 (-0.46, -0.12) |
| Kyrgyzstan | 72.62 | 59.95 | -0.77 (-0.84, -0.71) | 27.98 | 36.13 | 0.92 (0.85, 0.98) | 69.92 | 54.38 | -1.03 (-1.12, -0.94) |
| Mongolia | 56.07 | 51.04 | -0.19 (-0.28, -0.09) | 23.79 | 30.29 | 1.10 (0.98, 1.23) | 50.54 | 44.67 | -0.31 (-0.41, -0.22) |
| Tajikistan | 73.30 | 66.28 | -0.35 (-0.4, -0.31) | 26.29 | 29.56 | 0.51 (0.41, 0.61) | 68.18 | 57.68 | -0.58 (-0.60, -0.55) |
| Turkmenistan | 76.36 | 66.38 | -0.51 (-0.54, -0.48) | 26.47 | 35.66 | 1.32 (1.22, 1.41) | 73.97 | 59.62 | -0.84 (-0.93, -0.75) |
| Uzbekistan | 76.90 | 68.68 | -0.45 (-0.49, -0.41) | 28.41 | 35.69 | 0.89 (0.81, 0.98) | 71.66 | 59.51 | -0.81 (-0.89, -0.72) |
| Central Europe | 68.59 | 80.06 | 0.61 (0.54, 0.68) | 37.15 | 58.18 | 1.51 (1.32, 1.71) | 59.11 | 71.74 | 0.75 (0.71, 0.78) |
| Albania | 73.51 | 83.10 | 0.40 (0.30, 0.50) | 31.19 | 54.45 | 1.95 (1.87, 2.04) | 64.34 | 75.60 | 0.52 (0.38, 0.66) |
| Bosnia and Herzegovina | 60.67 | 68.53 | 0.57 (0.49, 0.65) | 32.72 | 51.19 | 1.87 (1.66, 2.07) | 50.88 | 61.29 | 0.83 (0.74, 0.92) |
| Bulgaria | 77.67 | 78.93 | 0.05 (-0.02, 0.12) | 35.93 | 46.54 | 0.92 (0.88, 0.97) | 68.36 | 68.91 | 0.01 (-0.08, 0.11) |
| Croatia | 78.89 | 89.68 | 0.58 (0.52, 0.65) | 56.57 | 74.00 | 0.97 (0.91, 1.02) | 71.12 | 83.46 | 0.69 (0.62, 0.75) |
| Czechia | 63.62 | 86.90 | 0.92 (0.78, 1.06) | 39.10 | 60.08 | 1.41 (1.25, 1.56) | 55.38 | 77.48 | 1.02 (0.88, 1.16) |
| Hungary | 56.54 | 79.60 | 1.12 (0.99, 1.24) | 40.28 | 59.90 | 1.30 (1.16, 1.44) | 49.93 | 72.48 | 1.23 (1.10, 1.36) |
| North Macedonia | 76.54 | 79.82 | 0.19 (0.12, 0.25) | 32.42 | 51.44 | 1.68 (1.56, 1.80) | 64.89 | 70.26 | 0.34 (0.27, 0.42) |
| Montenegro | 66.74 | 77.13 | 0.70 (0.52, 0.88) | 45.39 | 58.94 | 1.07 (0.96, 1.18) | 57.00 | 68.52 | 0.84 (0.68, 0.99) |
| Poland | 67.67 | 80.02 | 0.76 (0.62, 0.90) | 36.03 | 60.13 | 1.64 (1.22, 2.05) | 57.62 | 71.58 | 0.90 (0.85, 0.95) |
| Romania | 60.53 | 72.58 | 0.82 (0.74, 0.90) | 33.05 | 53.09 | 1.66 (1.57, 1.74) | 53.06 | 65.00 | 0.86 (0.80, 0.93) |
| Serbia | 81.50 | 78.89 | -0.16 (-0.21, -0.10) | 39.24 | 62.38 | 1.71 (1.64, 1.78) | 68.79 | 71.11 | 0.11 (0.05, 0.17) |
| Slovakia | 60.21 | 83.55 | 1.20 (1.14, 1.26) | 35.25 | 53.21 | 1.47 (1.42, 1.52) | 51.62 | 73.79 | 1.32 (1.25, 1.39) |
| Slovenia | 73.63 | 90.82 | 0.99 (0.89, 1.09) | 49.30 | 72.14 | 1.45 (1.38, 1.51) | 66.07 | 85.25 | 1.16 (1.06, 1.26) |
| **Eastern Europe** | 61.34 | 72.32 | 0.79 (0.59, 0.98) | 27.77 | 36.02 | 1.18 (1.08, 1.29) | 55.31 | 61.35 | 0.55 (0.34, 0.76) |
| Belarus | 61.37 | 78.70 | 0.96 (0.72, 1.19) | 37.36 | 56.41 | 1.61 (1.48, 1.74) | 58.91 | 71.63 | 0.74 (0.49, 1.00) |
| Estonia | 77.33 | 84.58 | 0.50 (0.21, 0.79) | 38.62 | 61.31 | 2.04 (1.88, 2.19) | 71.21 | 78.35 | 0.60 (0.29, 0.91) |
| Latvia | 68.05 | 75.93 | 0.53 (0.4, 0.65) | 41.80 | 56.81 | 1.34 (1.21, 1.48) | 63.36 | 69.22 | 0.50 (0.35, 0.66) |
| Lithuania | 66.07 | 68.51 | 0.10 (-0.05, 0.24) | 32.11 | 39.78 | 0.92 (0.82, 1.01) | 60.43 | 59.36 | -0.03 (-0.21, 0.14) |
| Republic of Moldova | 76.36 | 77.57 | 0.06 (-0.08, 0.19) | 31.61 | 42.25 | 1.12 (1.04, 1.20) | 71.53 | 68.29 | -0.14 (-0.30, 0.02) |
| Russian Federation | 57.74 | 73.58 | 1.12 (0.87, 1.37) | 24.12 | 27.84 | 0.64 (0.56, 0.71) | 49.26 | 60.47 | 0.98 (0.72, 1.25) |
| Ukraine | 62.08 | 67.28 | 0.53 (0.38, 0.67) | 34.03 | 44.34 | 1.22 (1.05, 1.40) | 58.88 | 59.73 | 0.23 (0.10, 0.36) |
| **High-income** | 81.27 | 89.26 | 0.31 (0.27, 0.35) | 69.37 | 80.57 | 0.51 (0.47, 0.55) | 78.10 | 86.22 | 0.33 (0.29, 0.36) |
| **High-income Asia Pacific** | 80.01 | 92.66 | 0.53 (0.48, 0.57) | 74.41 | 88.65 | 0.70 (0.66, 0.73) | 78.85 | 91.40 | 0.54 (0.50, 0.58) |
| Brunei Darussalam | 65.56 | 68.85 | 0.16 (0.14, 0.18) | 51.63 | 63.13 | 0.65 (0.60, 0.70) | 61.81 | 67.08 | 0.27 (0.25, 0.29) |
| Japan | 83.19 | 93.34 | 0.40 (0.35, 0.45) | 84.07 | 89.28 | 0.30 (0.24, 0.36) | 83.41 | 92.05 | 0.36 (0.33, 0.39) |
| Republic of Korea | 75.49 | 91.72 | 0.71 (0.67, 0.74) | 54.59 | 87.82 | 1.52 (1.33, 1.71) | 71.75 | 90.51 | 0.83 (0.79, 0.88) |
| Singapore | 69.27 | 91.72 | 1.14 (1.07, 1.21) | 70.47 | 87.34 | 0.80 (0.76, 0.83) | 69.60 | 90.56 | 1.04 (0.98, 1.10) |
| Australasia | 70.62 | 86.76 | 0.71 (0.63, 0.79) | 63.13 | 71.69 | 0.50 (0.47, 0.54) | 67.78 | 81.34 | 0.65 (0.58, 0.71) |
| Australia | 67.88 | 85.43 | 0.78 (0.69, 0.88) | 65.96 | 74.15 | 0.45 (0.42, 0.49) | 67.10 | 80.93 | 0.66 (0.59, 0.73) |
| New Zealand | 78.58 | 90.57 | 0.51 (0.47, 0.55) | 43.69 | 53.22 | 0.81 (0.72, 0.89) | 70.33 | 82.88 | 0.62 (0.58, 0.66) |
| **Western Europe** | 86.38 | 94.50 | 0.28 (0.23, 0.32) | 67.81 | 81.27 | 0.62 (0.57, 0.68) | 81.82 | 90.66 | 0.32 (0.27, 0.37) |
| Andorra | 86.78 | 93.92 | 0.22 (0.14, 0.29) | 78.61 | 86.27 | 0.29 (0.25, 0.33) | 83.31 | 90.83 | 0.25 (0.18, 0.32) |
| Austria | 83.54 | 93.55 | 0.33 (0.28, 0.39) | 68.70 | 79.78 | 0.47 (0.44, 0.51) | 78.29 | 89.19 | 0.39 (0.33, 0.45) |
| Belgium | 84.66 | 92.97 | 0.34 (0.30, 0.37) | 72.67 | 83.80 | 0.48 (0.46, 0.50) | 80.34 | 89.67 | 0.39 (0.35, 0.42) |
| Cyprus | 78.04 | 93.96 | 0.83 (0.75, 0.91) | 60.77 | 82.97 | 1.25 (1.14, 1.36) | 73.07 | 91.23 | 0.97 (0.88, 1.06) |
| Denmark | 82.37 | 93.90 | 0.46 (0.41, 0.50) | 83.25 | 92.51 | 0.39 (0.36, 0.43) | 82.67 | 93.24 | 0.43 (0.39, 0.47) |
| Finland | 80.13 | 94.17 | 0.59 (0.53, 0.66) | 82.21 | 90.94 | 0.34 (0.30, 0.38) | 81.15 | 92.74 | 0.48 (0.43, 0.53) |
| France | 85.07 | 94.57 | 0.39 (0.35, 0.42) | 71.01 | 84.33 | 0.61 (0.57, 0.65) | 81.44 | 91.18 | 0.41 (0.38, 0.45) |
| Germany | 88.88 | 95.83 | 0.17 (0.12, 0.23) | 52.80 | 71.81 | 0.98 (0.78, 1.18) | 81.31 | 90.00 | 0.23 (0.15, 0.30) |
| Greece | 87.46 | 93.14 | 0.23 (0.18, 0.27) | 75.77 | 82.48 | 0.29 (0.26, 0.32) | 83.32 | 89.40 | 0.25 (0.21, 0.30) |
| Iceland | 87.88 | 92.82 | 0.11 (0.05, 0.17) | 79.73 | 85.75 | 0.25 (0.19, 0.30) | 84.19 | 89.37 | 0.15 (0.09, 0.21) |
| Ireland | 80.19 | 94.43 | 0.60 (0.51, 0.69) | 71.21 | 86.49 | 0.75 (0.70, 0.80) | 76.64 | 91.39 | 0.66 (0.57, 0.75) |
| Israel | 73.91 | 89.41 | 0.73 (0.66, 0.79) | 63.68 | 78.91 | 0.76 (0.73, 0.79) | 70.26 | 85.10 | 0.71 (0.66, 0.77) |
| Italy | 87.83 | 94.90 | 0.24 (0.20, 0.29) | 65.53 | 76.55 | 0.52 (0.48, 0.57) | 83.67 | 91.52 | 0.28 (0.22, 0.34) |
| Luxembourg | 86.60 | 92.29 | 0.21 (0.16, 0.26) | 70.46 | 84.21 | 0.57 (0.47, 0.66) | 82.03 | 89.35 | 0.28 (0.23, 0.34) |
| Malta | 77.12 | 91.92 | 0.59 (0.52, 0.67) | 69.58 | 81.12 | 0.53 (0.50, 0.56) | 74.25 | 88.30 | 0.59 (0.52, 0.65) |
| Netherlands | 89.33 | 95.01 | 0.20 (0.17, 0.23) | 78.78 | 86.89 | 0.39 (0.36, 0.42) | 86.38 | 92.37 | 0.23 (0.20, 0.25) |
| Norway | 85.88 | 92.94 | 0.26 (0.23, 0.29) | 85.45 | 92.07 | 0.25 (0.21, 0.28) | 85.69 | 92.45 | 0.25 (0.22, 0.29) |
| Portugal | 76.49 | 91.34 | 0.71 (0.65, 0.78) | 55.04 | 77.12 | 1.13 (1.06, 1.19) | 71.42 | 87.39 | 0.77 (0.71, 0.83) |
| Spain | 84.93 | 95.04 | 0.38 (0.31, 0.44) | 68.89 | 83.62 | 0.66 (0.60, 0.71) | 81.64 | 92.43 | 0.42 (0.35, 0.48) |
| Sweden | 89.53 | 93.97 | 0.10 (0.07, 0.13) | 85.82 | 91.49 | 0.19 (0.16, 0.22) | 88.09 | 92.91 | 0.13 (0.10, 0.16) |
| Switzerland | 89.47 | 94.78 | 0.14 (0.09, 0.18) | 80.00 | 86.41 | 0.25 (0.23, 0.27) | 87.90 | 91.85 | 0.10 (0.07, 0.13) |
| United Kingdom | 86.50 | 93.69 | 0.25 (0.21, 0.28) | 61.62 | 74.02 | 0.70 (0.66, 0.73) | 80.70 | 88.40 | 0.28 (0.24, 0.33) |
| **Southern Latin America** | 61.45 | 66.46 | 0.28 (0.23, 0.33) | 41.59 | 56.19 | 0.98 (0.94, 1.02) | 57.53 | 63.49 | 0.34 (0.29, 0.40) |
| Argentina | 62.15 | 62.91 | 0.00 (-0.03, 0.04) | 40.42 | 52.41 | 0.76 (0.7, 0.83) | 57.87 | 59.80 | 0.06 (0.01, 0.10) |
| Chile | 58.30 | 73.77 | 1.00 (0.89, 1.10) | 43.35 | 65.84 | 1.53 (1.48, 1.59) | 55.43 | 71.68 | 1.07 (0.98, 1.16) |
| Uruguay | 66.41 | 70.82 | 0.24 (0.21, 0.27) | 46.82 | 57.62 | 0.73 (0.69, 0.76) | 61.95 | 66.41 | 0.24 (0.21, 0.26) |
| **High-income North America** | 77.77 | 80.88 | 0.15 (0.13, 0.17) | 72.66 | 80.66 | 0.32 (0.27, 0.37) | 75.94 | 80.80 | 0.21 (0.19, 0.23) |
| Canada | 88.04 | 93.57 | 0.22 (0.20, 0.24) | 77.55 | 85.07 | 0.37 (0.35, 0.39) | 84.80 | 90.51 | 0.25 (0.22, 0.27) |
| United States of America | 75.97 | 77.79 | 0.10 (0.08, 0.13) | 72.02 | 80.10 | 0.31 (0.25, 0.37) | 74.53 | 79.02 | 0.20 (0.17, 0.22) |
| **Latin America and Caribbean** | 66.86 | 61.96 | -0.32 (-0.34, -0.30) | 34.21 | 47.24 | 1.07 (1.02, 1.11) | 62.37 | 58.39 | -0.28 (-0.31, -0.25) |
| Caribbean | 69.93 | 68.07 | -0.11 (-0.13, -0.09) | 32.13 | 38.31 | 0.58 (0.46, 0.70) | 67.31 | 63.25 | -0.24 (-0.28, -0.20) |
| Antigua and Barbuda | 74.37 | 72.21 | 0.01 (-0.05, 0.07) | 45.60 | 50.44 | 0.48 (0.35, 0.61) | 72.19 | 67.33 | -0.15 (-0.22, -0.07) |
| Bahamas | 59.02 | 55.71 | -0.27 (-0.32, -0.22) | 38.02 | 43.25 | 0.54 (0.44, 0.64) | 56.48 | 52.16 | -0.34 (-0.38, -0.31) |
| Barbados | 66.94 | 65.89 | 0.01 (-0.02, 0.04) | 41.93 | 51.56 | 0.66 (0.61, 0.71) | 65.31 | 62.67 | -0.10 (-0.14, -0.07) |
| Belize | 79.17 | 70.86 | -0.36 (-0.39, -0.34) | 32.89 | 38.70 | 0.62 (0.53, 0.71) | 76.68 | 64.08 | -0.63 (-0.67, -0.58) |
| Cuba | 57.77 | 66.74 | 0.58 (0.52, 0.63) | 49.30 | 60.36 | 0.80 (0.71, 0.89) | 56.91 | 64.44 | 0.50 (0.42, 0.57) |
| Dominica | 61.47 | 60.54 | -0.13 (-0.16, -0.09) | 37.16 | 38.09 | 0.14 (0.05, 0.23) | 60.22 | 57.98 | -0.21 (-0.25, -0.17) |
| Dominican Republic | 77.47 | 70.02 | -0.46 (-0.51, -0.42) | 28.51 | 38.39 | 0.90 (0.78, 1.01) | 74.92 | 66.96 | -0.52 (-0.58, -0.46) |
| Grenada | 72.52 | 71.31 | 0.03 (0.00, 0.07) | 32.66 | 41.58 | 0.60 (0.53, 0.67) | 70.25 | 65.11 | -0.21 (-0.26, -0.16) |
| Guyana | 61.46 | 55.03 | -0.42 (-0.47, -0.37) | 27.21 | 32.11 | 0.35 (0.22, 0.48) | 59.20 | 51.13 | -0.56 (-0.61, -0.50) |
| Haiti | 70.79 | 68.04 | -0.14 (-0.17, -0.12) | 21.74 | 23.33 | 0.20 (0.11, 0.28) | 67.75 | 62.35 | -0.31 (-0.35, -0.26) |
| Jamaica | 71.62 | 61.42 | -0.60 (-0.66, -0.55) | 39.84 | 43.49 | 0.37 (0.30, 0.44) | 69.10 | 57.57 | -0.70 (-0.77, -0.62) |
| Saint Lucia | 67.15 | 65.08 | -0.11 (-0.14, -0.08) | 34.76 | 44.86 | 0.93 (0.88, 0.98) | 65.19 | 61.05 | -0.24 (-0.26, -0.22) |
| Saint Vincent and the Grenadines | 75.62 | 66.37 | -0.47 (-0.51, -0.43) | 34.90 | 38.80 | 0.40 (0.32, 0.47) | 73.57 | 61.75 | -0.63 (-0.70, -0.57) |
| Suriname | 71.07 | 68.45 | -0.14 (-0.16, -0.12) | 30.16 | 35.04 | 0.57 (0.43, 0.71) | 67.28 | 58.23 | -0.54 (-0.62, -0.47) |
| Trinidad and Tobago | 54.21 | 56.26 | 0.26 (0.18, 0.33) | 32.81 | 41.41 | 1.01 (0.91, 1.11) | 52.67 | 52.60 | 0.09 (0.03, 0.15) |
| **Andean Latin America** | 66.32 | 63.37 | -0.22 (-0.27, -0.16) | 27.25 | 43.01 | 1.69 (1.64, 1.75) | 61.30 | 59.24 | -0.18 (-0.23, -0.12) |
| Bolivia (Plurinational State of) | 66.62 | 63.20 | -0.21 (-0.24, -0.18) | 23.12 | 32.47 | 1.16 (1.11, 1.22) | 63.09 | 57.89 | -0.34 (-0.36, -0.31) |
| Ecuador | 62.76 | 57.01 | -0.52 (-0.66, -0.39) | 26.71 | 43.01 | 1.61 (1.51, 1.70) | 60.67 | 54.38 | -0.58 (-0.69, -0.47) |
| Peru | 67.53 | 67.01 | -0.05 (-0.10, 0.00) | 28.14 | 46.87 | 1.91 (1.84, 1.98) | 60.73 | 62.48 | 0.09 (0.03, 0.15) |
| **Central Latin America** | 66.55 | 60.52 | -0.41 (-0.45, -0.38) | 34.86 | 49.06 | 1.08 (1.01, 1.16) | 63.33 | 58.42 | -0.36 (-0.40, -0.32) |
| Colombia | 66.63 | 64.35 | -0.17 (-0.17, -0.07) | 34.31 | 53.92 | 1.52 (1.44, 1.61) | 62.75 | 62.18 | -0.06 (-0.18, 0.05) |
| Costa Rica | 53.72 | 63.49 | 0.68 (0.62, 0.75) | 47.28 | 59.30 | 0.90 (0.83, 0.97) | 52.85 | 62.42 | 0.69 (0.62, 0.76) |
| El Salvador | 80.72 | 74.49 | -0.46 (-0.46, -0.46) | 28.50 | 45.93 | 1.65 (1.54, 1.75) | 78.01 | 69.65 | -0.59 (-0.70, -0.48) |
| Guatemala | 78.57 | 66.65 | -0.73 (-0.86, -0.6) | 24.21 | 34.97 | 1.31 (1.21, 1.41) | 75.29 | 62.94 | -0.81 (-0.92, -0.69) |
| Honduras | 67.28 | 62.84 | -0.26 (-0.31, -0.21) | 25.89 | 35.14 | 1.03 (0.98, 1.08) | 63.57 | 57.94 | -0.35 (-0.40, -0.29) |
| Mexico | 66.28 | 57.88 | -0.55 (-0.59, -0.5) | 36.28 | 49.28 | 0.89 (0.79, 0.99) | 62.95 | 56.33 | -0.46 (-0.51, -0.41) |
| Nicaragua | 63.02 | 57.09 | -0.42 (-0.49, -0.34) | 30.06 | 47.59 | 1.81 (1.69, 1.93) | 59.74 | 55.18 | -0.33 (-0.45, -0.21) |
| Panama | 50.03 | 58.53 | 0.39 (0.34, 0.45) | 38.71 | 52.67 | 0.84 (0.72, 0.96) | 48.16 | 57.03 | 0.44 (0.36, 0.52) |
| Venezuela (Bolivarian Republic of) | 55.47 | 54.95 | -0.06 (-0.13, 0.00) | 34.09 | 48.45 | 1.28 (1.14, 1.41) | 54.40 | 53.72 | -0.05 (-0.11, 0.01) |
| **Tropical Latin America** | 66.53 | 61.23 | -0.3 (-0.33, -0.28) | 35.36 | 48.20 | 1.03 (0.99, 1.06) | 60.22 | 56.41 | -0.24 (-0.29, -0.18) |
| Brazil | 66.27 | 61.19 | -0.28 (-0.31, -0.25) | 35.40 | 48.28 | 1.03 (0.99, 1.06) | 59.90 | 56.33 | -0.21 (-0.27, -0.16) |
| Paraguay | 74.02 | 62.31 | -0.81 (-0.9, -0.73) | 31.42 | 42.72 | 0.96 (0.85, 1.08) | 71.00 | 58.84 | -0.87 (-0.95, -0.79) |
| **North Africa and Middle East** | 72.31 | 71.89 | 0.00 (-0.01, 0.02) | 42.29 | 67.17 | 1.60 (1.53, 1.67) | 67.10 | 70.29 | 0.17 (0.15, 0.20) |
| Algeria | 64.43 | 66.09 | 0.11 (0.08, 0.14) | 43.88 | 72.04 | 1.70 (1.65, 1.75) | 60.26 | 68.67 | 0.47 (0.42, 0.52) |
| Bahrain | 51.69 | 65.19 | 0.96 (0.87, 1.05) | 48.52 | 81.16 | 1.97 (1.84, 2.10) | 50.88 | 72.18 | 1.41 (1.32, 1.50) |
| Egypt | 67.34 | 65.29 | -0.04 (-0.08, 0.01) | 34.81 | 60.42 | 1.76 (1.65, 1.88) | 61.23 | 63.50 | 0.15 (0.09, 0.21) |
| Iran (Islamic Republic of) | 72.16 | 71.34 | -0.05 (-0.13, 0.03) | 51.22 | 79.07 | 1.30 (1.14, 1.45) | 67.92 | 74.71 | 0.28 (0.21, 0.36) |
| Iraq | 80.73 | 80.95 | 0.05 (0.03, 0.07) | 41.32 | 66.23 | 1.90 (1.72, 2.08) | 74.16 | 75.50 | 0.10 (0.06, 0.13) |
| Jordan | 84.53 | 88.69 | 0.18 (0.18, 0.19) | 45.88 | 77.00 | 2.03 (1.93, 2.12) | 79.80 | 85.92 | 0.27 (0.26, 0.28) |
| Kuwait | 83.97 | 86.80 | 0.11 (0.02, 0.19) | 70.37 | 88.51 | 0.83 (0.79, 0.88) | 81.77 | 87.46 | 0.24 (0.17, 0.32) |
| Lebanon | 67.01 | 80.99 | 0.76 (0.70, 0.82) | 52.87 | 87.25 | 1.81 (1.67, 1.95) | 63.94 | 83.58 | 1.05 (1.01, 1.10) |
| Libya | 70.72 | 67.97 | 0.03 (-0.06, 0.11) | 48.83 | 68.47 | 1.31 (1.16, 1.46) | 65.57 | 68.20 | 0.32 (0.22, 0.42) |
| Morocco | 66.70 | 63.31 | -0.03 (-0.08, 0.03) | 36.23 | 57.70 | 1.64 (1.56, 1.71) | 58.98 | 60.84 | 0.19 (0.13, 0.26) |
| Palestine | 84.85 | 83.29 | -0.05 (-0.07, -0.04) | 49.13 | 68.38 | 1.09 (0.98, 1.19) | 76.84 | 77.10 | 0.01 (-0.02, 0.04) |
| Oman | 69.70 | 75.47 | 0.31 (0.23, 0.39) | 51.30 | 82.90 | 1.48 (1.28, 1.68) | 66.33 | 78.50 | 0.62 (0.58, 0.65) |
| Qatar | 66.53 | 76.64 | 0.61 (0.54, 0.68) | 52.15 | 87.08 | 2.02 (1.83, 2.22) | 62.65 | 81.99 | 1.14 (1.06, 1.22) |
| Saudi Arabia | 59.57 | 69.01 | 0.43 (0.32, 0.54) | 41.41 | 81.36 | 2.27 (2.17, 2.38) | 55.26 | 75.60 | 1.06 (1.01, 1.10) |
| Syrian Arab Republic | 77.18 | 77.22 | 0.08 (0.04, 0.11) | 42.88 | 70.93 | 1.87 (1.69, 2.06) | 74.28 | 75.88 | 0.14 (0.10, 0.17) |
| Tunisia | 70.44 | 70.04 | -0.02 (-0.11, 0.06) | 51.30 | 79.94 | 1.57 (1.48, 1.65) | 67.81 | 73.63 | 0.31 (0.23, 0.40) |
| Turkey | 69.74 | 72.24 | 0.09 (0.04, 0.14) | 40.92 | 80.49 | 2.63 (2.41, 2.85) | 64.61 | 76.02 | 0.62 (0.55, 0.68) |
| United Arab Emirates | 59.23 | 57.03 | 0.01 (-0.06, 0.07) | 44.59 | 65.68 | 1.23 (1.09, 1.36) | 52.61 | 62.68 | 0.64 (0.58, 0.69) |
| Yemen | 75.11 | 70.38 | -0.14 (-0.17, -0.1) | 31.32 | 43.45 | 1.45 (1.35, 1.56) | 69.32 | 64.50 | -0.14 (-0.18, -0.10) |
| **South Asia** | 58.02 | 57.64 | 0.06 (0.03, 0.09) | 23.47 | 35.26 | 1.55 (1.44, 1.65) | 50.45 | 50.23 | 0.05 (0.01, 0.09) |
| Afghanistan | 69.12 | 67.05 | -0.14 (-0.15, -0.12) | 25.67 | 31.22 | 0.73 (0.56, 0.90) | 63.00 | 60.40 | -0.17 (-0.19, -0.14) |
| Bangladesh | 58.81 | 51.64 | -0.45 (-0.47, -0.42) | 22.55 | 31.98 | 1.14 (1.01, 1.27) | 51.39 | 44.56 | -0.52 (-0.56, -0.49) |
| Bhutan | 61.26 | 58.35 | -0.13 (-0.18, -0.09) | 23.84 | 35.39 | 1.49 (1.38, 1.59) | 53.09 | 50.06 | -0.18 (-0.21, -0.15) |
| India | 56.28 | 55.43 | -0.01 (-0.03, 0.02) | 22.30 | 35.42 | 1.67 (1.54, 1.80) | 48.72 | 48.25 | -0.03 (-0.06, 0.01) |
| Nepal | 60.39 | 52.25 | -0.52 (-0.58, -0.47) | 23.35 | 30.36 | 0.87 (0.80, 0.94) | 52.52 | 44.18 | -0.66 (-0.69, -0.63) |
| Pakistan | 65.19 | 62.45 | 0.01 (-0.08, 0.10) | 30.25 | 36.11 | 0.95 (0.74, 1.15) | 57.60 | 55.22 | 0.04 (-0.07, 0.14) |
| **Sub-Saharan Africa** | 64.76 | 66.28 | 0.14 (0.10, 0.17) | 23.50 | 25.98 | 0.34 (0.28, 0.39) | 60.95 | 60.18 | 0.01 (-0.02, 0.04) |
| **Central Sub-Saharan Africa** | 73.68 | 71.49 | -0.07 (-0.11, -0.04) | 22.97 | 24.01 | 0.12 (0.06, 0.19) | 69.26 | 65.21 | -0.18 (-0.21, -0.14) |
| Angola | 73.08 | 72.05 | -0.04 (-0.06, -0.01) | 22.18 | 24.55 | 0.33 (0.26, 0.39) | 69.01 | 65.73 | -0.16 (-0.19, -0.14) |
| Central African Republic | 72.01 | 70.70 | -0.01 (-0.03, 0.01) | 21.26 | 21.03 | -0.01 (-0.04, 0.03) | 67.62 | 65.06 | -0.07 (-0.10, -0.05) |
| Congo | 71.72 | 68.99 | -0.06 (-0.10, -0.02) | 23.24 | 25.63 | 0.39 (0.30, 0.49) | 67.29 | 62.31 | -0.20 (-0.25, -0.15) |
| Democratic Republic of the Congo | 74.12 | 71.48 | -0.09 (-0.13, -0.05) | 23.27 | 23.71 | 0.02 (-0.04, 0.08) | 69.59 | 65.18 | -0.19 (-0.23, -0.14) |
| Equatorial Guinea | 71.14 | 68.08 | -0.12 (-0.20, -0.04) | 20.82 | 30.59 | 1.47 (1.40, 1.55) | 66.70 | 61.98 | -0.23 (-0.32, -0.14) |
| Gabon | 72.03 | 68.08 | -0.17 (-0.19, -0.14) | 24.98 | 29.68 | 0.58 (0.47, 0.69) | 67.14 | 61.55 | -0.28 (-0.3, -0.26) |
| **Eastern Sub-Saharan Africa** | 63.87 | 67.47 | 0.25 (0.22, 0.28) | 22.64 | 25.54 | 0.42 (0.36, 0.47) | 61.01 | 62.68 | 0.15 (0.12, 0.19) |
| Burundi | 69.78 | 71.22 | 0.14 (0.09, 0.19) | 21.80 | 23.31 | 0.36 (0.29, 0.43) | 64.50 | 64.92 | 0.11 (0.05, 0.16) |
| Comoros | 72.04 | 70.64 | -0.02 (-0.07, 0.04) | 22.38 | 24.91 | 0.40 (0.32, 0.47) | 67.57 | 64.17 | -0.11 (-0.18, -0.04) |
| Djibouti | 73.97 | 73.27 | 0.01 (-0.01, 0.03) | 23.65 | 26.72 | 0.47 (0.35, 0.60) | 69.67 | 67.45 | -0.06 (-0.08, -0.04) |
| Eritrea | 69.52 | 69.43 | -0.01 (-0.02, 0.00) | 20.16 | 23.61 | 0.45 (0.42, 0.49) | 65.18 | 63.16 | -0.11 (-0.12, -0.10) |
| Ethiopia | 56.28 | 58.45 | 0.21 (0.18, 0.24) | 21.27 | 27.14 | 0.94 (0.87, 1.01) | 54.56 | 55.95 | 0.17 (0.14, 0.20) |
| Kenya | 72.10 | 68.89 | -0.16 (-0.16, -0.15) | 26.13 | 26.53 | -0.15 (-0.30, -0.01) | 66.83 | 61.09 | -0.32 (-0.33, -0.31) |
| Madagascar | 71.66 | 69.68 | -0.14 (-0.18, -0.09) | 22.71 | 23.38 | -0.03 (-0.10, 0.04) | 66.92 | 63.34 | -0.23 (-0.28, -0.19) |
| Malawi | 69.96 | 66.64 | -0.14 (-0.17, -0.10) | 23.65 | 24.76 | 0.14 (0.09, 0.20) | 64.70 | 58.91 | -0.30 (-0.35, -0.26) |
| Mauritius | 82.37 | 77.34 | -0.33 (-0.40, -0.27) | 44.01 | 54.37 | 0.53 (0.44, 0.61) | 79.52 | 74.35 | -0.36 (-0.42, -0.30) |
| Mozambique | 72.73 | 73.12 | -0.01 (-0.04, 0.02) | 21.88 | 23.59 | 0.17 (0.12, 0.21) | 68.65 | 67.58 | -0.09 (-0.12, -0.06) |
| Rwanda | 68.34 | 71.39 | 0.27 (0.21, 0.32) | 21.30 | 26.47 | 1.03 (0.77, 1.30) | 63.06 | 64.81 | 0.22 (0.16, 0.28) |
| Seychelles | 61.01 | 62.23 | 0.19 (0.09, 0.29) | 37.52 | 49.88 | 1.03 (0.97, 1.09) | 55.81 | 58.72 | 0.29 (0.20, 0.38) |
| Somalia | 71.90 | 71.08 | 0.00 (-0.02, 0.01) | 21.51 | 21.07 | -0.04 (-0.12, 0.04) | 67.94 | 65.68 | -0.08 (-0.09, -0.06) |
| United Republic of Tanzania | 74.05 | 73.51 | -0.03 (-0.06, -0.01) | 24.42 | 26.20 | 0.17 (0.11, 0.23) | 69.63 | 67.54 | -0.11 (-0.14, -0.08) |
| Uganda | 72.70 | 75.09 | 0.17 (0.14, 0.20) | 23.98 | 25.92 | 0.30 (0.20, 0.40) | 68.52 | 67.98 | 0.03 (0.00, 0.06) |
| Zambia | 71.50 | 70.18 | -0.04 (-0.06, -0.02) | 22.86 | 26.54 | 0.54 (0.41, 0.67) | 67.45 | 64.47 | -0.14 (-0.15, -0.12) |
| **Southern Sub-Saharan Africa** | 74.97 | 72.99 | -0.17 (-0.26, -0.09) | 27.91 | 32.45 | 0.55 (0.32, 0.78) | 68.66 | 64.85 | -0.29 (-0.39, -0.2) |
| Botswana | 70.60 | 74.43 | 0.26 (0.19, 0.32) | 27.57 | 35.34 | 0.77 (0.61, 0.93) | 63.94 | 67.12 | 0.24 (0.17, 0.31) |
| Lesotho | 71.52 | 68.78 | -0.16 (-0.22, -0.10) | 23.59 | 24.07 | -0.07 (-0.16, 0.02) | 64.89 | 61.59 | -0.19 (-0.26, -0.12) |
| Namibia | 71.21 | 72.27 | 0.11 (0.04, 0.19) | 24.75 | 31.26 | 0.76 (0.58, 0.93) | 62.82 | 61.74 | 0.01 (-0.08, 0.09) |
| **South Africa** | 76.09 | 77.50 | -0.01 (-0.12, 0.10) | 28.16 | 35.45 | 0.95 (0.65, 1.24) | 69.75 | 68.80 | -0.15 (-0.27, -0.03) |
| Eswatini | 72.10 | 69.79 | -0.11 (-0.2, -0.03) | 24.54 | 26.60 | 0.14 (0.00, 0.28) | 65.85 | 62.41 | -0.18 (-0.27, -0.10) |
| Zimbabwe | 71.71 | 61.35 | -0.61 (-0.7, -0.53) | 28.30 | 24.73 | -0.99 (-1.20, -0.78) | 65.86 | 54.39 | -0.74 (-0.83, -0.65) |
| **Western Sub-Saharan Africa** | 59.48 | 61.30 | 0.19 (0.15, 0.24) | 24.27 | 26.28 | 0.26 (0.21, 0.32) | 53.28 | 53.50 | 0.10 (0.06, 0.14) |
| Benin | 61.20 | 62.78 | 0.17 (0.13, 0.21) | 24.00 | 25.44 | 0.16 (0.13, 0.19) | 56.56 | 56.55 | 0.09 (0.04, 0.14) |
| Burkina Faso | 62.55 | 66.67 | 0.32 (0.26, 0.37) | 23.86 | 25.13 | 0.18 (0.14, 0.22) | 57.68 | 60.07 | 0.24 (0.19, 0.29) |
| Cameroon | 61.16 | 62.89 | 0.17 (0.12, 0.22) | 24.96 | 26.57 | 0.11 (-0.01, 0.22) | 56.59 | 56.35 | 0.05 (0.00, 0.10) |
| Cabo Verde | 67.26 | 65.50 | -0.08 (-0.1, -0.06) | 29.21 | 41.83 | 1.45 (1.29, 1.61) | 62.55 | 58.31 | -0.25 (-0.30, -0.20) |
| Chad | 61.68 | 63.70 | 0.19 (0.15, 0.23) | 23.38 | 22.94 | -0.07 (-0.11, -0.02) | 57.05 | 57.52 | 0.11 (0.06, 0.15) |
| Côte d'Ivoire | 59.32 | 60.17 | 0.19 (0.12, 0.25) | 24.78 | 25.47 | 0.13 (0.05, 0.20) | 54.38 | 53.65 | 0.1 (0.03, 0.17) |
| Gambia | 57.33 | 52.86 | -0.28 (-0.29, -0.26) | 24.79 | 26.08 | 0.13 (0.05, 0.22) | 53.27 | 46.10 | -0.53 (-0.54, -0.51) |
| Ghana | 46.03 | 48.71 | 0.38 (0.26, 0.51) | 26.42 | 29.39 | 0.23 (0.15, 0.32) | 41.19 | 41.53 | 0.13 (0.06, 0.19) |
| Guinea | 53.58 | 51.63 | -0.08 (-0.11, -0.06) | 22.79 | 23.18 | 0.05 (0.02, 0.07) | 49.35 | 45.33 | -0.29 (-0.30, -0.27) |
| Guinea-Bissau | 58.10 | 58.39 | 0.13 (0.08, 0.19) | 22.49 | 23.41 | 0.27 (0.18, 0.36) | 53.86 | 52.01 | 0.00 (-0.06, 0.06) |
| Liberia | 60.53 | 59.4 | -0.01 (-0.09, 0.06) | 23.10 | 25.06 | 0.29 (0.24, 0.33) | 55.74 | 52.90 | -0.12 (-0.19, -0.05) |
| Mali | 56.84 | 56.01 | -0.04 (-0.09, 0.01) | 22.90 | 24.66 | 0.23 (0.20, 0.26) | 53.26 | 48.94 | -0.34 (-0.37, -0.31) |
| Mauritania | 56.85 | 59.13 | 0.24 (0.17, 0.30) | 23.62 | 28.29 | 0.61 (0.53, 0.69) | 52.48 | 52.96 | 0.13 (0.07, 0.18) |
| Niger | 65.68 | 67.97 | 0.20 (0.16, 0.23) | 22.79 | 24.15 | 0.18 (0.15, 0.21) | 61.29 | 61.80 | 0.10 (0.07, 0.14) |
| Nigeria | 60.22 | 59.75 | 0.04 (0.00, 0.08) | 24.10 | 26.91 | 0.40 (0.33, 0.47) | 52.00 | 50.60 | -0.03 (-0.07, 0.01) |
| Sao Tome and Principe | 59.09 | 59.31 | -0.01 (-0.07, 0.04) | 23.19 | 30.41 | 0.83 (0.68, 0.99) | 56.74 | 56.00 | -0.08 (-0.13, -0.02) |
| Senegal | 59.32 | 59.66 | 0.09 (0.05, 0.14) | 24.09 | 25.75 | 0.20 (0.14, 0.27) | 54.75 | 53.09 | -0.04 (-0.08, 0.00) |
| Sierra Leone | 61.50 | 64.20 | 0.26 (0.20, 0.32) | 22.81 | 24.84 | 0.30 (0.22, 0.37) | 56.40 | 57.39 | 0.18 (0.12, 0.25) |
| Togo | 60.55 | 60.42 | 0.10 (0.05, 0.16) | 24.94 | 26.14 | 0.16 (0.08, 0.25) | 55.58 | 53.93 | 0.02 (-0.04, 0.07) |
| American Samoa | 68.10 | 68.58 | 0.10 (0.06, 0.14) | 37.97 | 40.39 | 0.26 (0.18, 0.34) | 61.08 | 60.54 | 0.03 (0.00, 0.07) |
| Bermuda | 71.98 | 81.52 | 0.52 (0.48, 0.55) | 52.32 | 72.56 | 1.20 (1.11, 1.28) | 69.88 | 78.52 | 0.48 (0.44, 0.52) |
| Cook Islands | 74.23 | 69.54 | -0.28 (-0.31, -0.26) | 48.78 | 59.92 | 0.69 (0.64, 0.74) | 68.75 | 65.38 | -0.28 (-0.31, -0.24) |
| Greenland | 48.04 | 56.52 | 0.64 (0.58, 0.71) | 37.72 | 48.21 | 0.92 (0.87, 0.96) | 42.07 | 51.33 | 0.74 (0.69, 0.79) |
| Guam | 69.70 | 78.25 | 0.45 (0.41, 0.50) | 49.62 | 52.67 | 0.11 (-0.03, 0.26) | 66.79 | 72.32 | 0.30 (0.26, 0.33) |
| Monaco | 92.65 | 93.06 | 0.01 (-0.01, 0.02) | 81.57 | 86.01 | 0.20 (0.18, 0.21) | 90.62 | 91.34 | 0.02 (0.01, 0.03) |
| Nauru | 75.51 | 75.42 | 0.01 (-0.01, 0.03) | 35.52 | 39.93 | 0.29 (-0.05, 0.63) | 70.39 | 69.57 | -0.04 (-0.06, -0.02) |
| Niue | 73.40 | 75.74 | 0.18 (0.14, 0.21) | 40.41 | 52.98 | 1.06 (0.99, 1.13) | 67.70 | 70.80 | 0.22 (0.19, 0.26) |
| Northern Mariana Islands | 67.44 | 74.83 | 0.32 (0.27, 0.38) | 55.06 | 60.83 | 0.07 (-0.04, 0.19) | 64.67 | 70.22 | 0.21 (0.13, 0.28) |
| Palau | 69.23 | 68.3 | -0.04 (-0.06, -0.03) | 45.20 | 52.71 | 0.44 (0.41, 0.47) | 60.43 | 61.68 | 0.05 (0.04, 0.06) |
| Puerto Rico | 62.33 | 77.31 | 0.93 (0.85, 1.00) | 51.05 | 66.81 | 1.11 (1.03, 1.18) | 60.83 | 74.25 | 0.89 (0.81, 0.97) |
| Saint Kitts and Nevis | 78.06 | 77.55 | -0.04 (-0.06, -0.02) | 40.00 | 52.32 | 0.72 (0.65, 0.80) | 76.30 | 73.16 | -0.21 (-0.23, -0.18) |
| San Marino | 92.91 | 97.07 | 0.14 (0.11, 0.16) | 79.49 | 86.05 | 0.29 (0.27, 0.30) | 89.19 | 94.05 | 0.17 (0.14, 0.20) |
| Tokelau | 74.41 | 72.40 | -0.11 (-0.13, -0.09) | 31.78 | 42.90 | 1.09 (1.00, 1.18) | 68.62 | 66.09 | -0.14 (-0.17, -0.12) |
| Tuvalu | 75.58 | 69.79 | -0.28 (-0.29, -0.27) | 28.05 | 35.35 | 0.70 (0.66, 0.74) | 70.35 | 63.60 | -0.37 (-0.39, -0.36) |
| United States Virgin Islands | 65.27 | 66.56 | 0.15 (0.09, 0.20) | 39.96 | 48.51 | 0.78 (0.68, 0.88) | 61.30 | 61.01 | 0.04 (-0.01, 0.09) |
| South Sudan | 72.91 | 71.75 | -0.03 (-0.06, 0.00) | 22.67 | 22.86 | 0.08 (0.04, 0.13) | 69.71 | 67.23 | -0.09 (-0.12, -0.06) |
| Sudan | 75.51 | 70.65 | -0.24 (-0.28, -0.21) | 32.19 | 49.46 | 1.37 (1.29, 1.46) | 69.63 | 65.46 | -0.25 (-0.28, -0.22) |
| **High SDI** | 81.35 | 89.63 | 0.32 (0.29, 0.35) | 69.52 | 81.48 | 0.56 (0.52, 0.60) | 72.48 | 80.77 | 0.35 (0.32, 0.38) |
| **High-middle SDI** | 77.80 | 84.72 | 0.27 (0.24, 0.30) | 40.25 | 70.01 | 2.13 (2.01, 2.25) | 78.14 | 86.61 | 0.36 (0.33, 0.39) |
| **Middle SDI** | 77.38 | 76.64 | -0.13 (-0.16, -0.09) | 33.45 | 60.71 | 2.09 (2.04, 2.14) | 62.91 | 58.63 | -0.07 (-0.12, -0.03) |
| **Low-middle SDI** | 69.34 | 65.80 | -0.19 (-0.20, -0.18) | 25.32 | 37.43 | 1.45 (1.36, 1.54) | 59.38 | 59.42 | -0.26 (-0.28, -0.25) |
| **Low SDI** | 64.01 | 65.75 | 0.16 (0.13, 0.18) | 23.24 | 29.72 | 0.88 (0.82, 0.95) | 72.24 | 72.66 | 0.07 (0.04, 0.10) |

Table 3：

Quality of Care Index (QCI) for girls and boys, and gender disparity ratio (GDR) in different age groups of global, and 5 SDI regions in 1990 and 2019

|  |  | **Leukemia** | | | | | | | | **Brain and central nervous system cancer** | | | | | | | |
| --- | --- | --- | --- | --- | --- | --- | --- | --- | --- | --- | --- | --- | --- | --- | --- | --- | --- |
|  |  | **2019** | | | | **1990** | | | | **2019** | | | | **1990** | | | |
| **Age groups** | **Regions** | **QCI** | **Girls' QCI** | **Boy's QCI** | **GDR** | **QCI** | **Girls' QCI** | **Boy's QCI** | **GDR** | **QCI** | **Girls' QCI** | **Boy's QCI** | **GDR** | **QCI** | **Girls' QCI** | **Boy's QCI** | **GDR** |
| <20 years | Global | 74.71 | 78.00 | 71.80 | 1.09 | 74.68 | 78.02 | 71.01 | 1.10 | 56.59 | 61.17 | 52.71 | 1.16 | 38.02 | 41.01 | 35.75 | 1.15 |
|  | High SDI | 89.63 | 90.45 | 88.93 | 1.02 | 81.35 | 84.38 | 78.38 | 1.08 | 81.48 | 82.17 | 80.92 | 1.02 | 69.52 | 70.95 | 68.29 | 1.04 |
|  | High-middle SDI | 84.72 | 86.50 | 83.19 | 1.04 | 77.80 | 80.61 | 74.98 | 1.08 | 70.01 | 74.53 | 65.43 | 1.14 | 40.25 | 43.37 | 37.58 | 1.15 |
|  | Middle SDI | 76.64 | 78.78 | 74.95 | 1.05 | 77.38 | 79.99 | 74.62 | 1.07 | 60.17 | 65.50 | 54.73 | 1.20 | 33.45 | 36.40 | 31.04 | 1.17 |
|  | Low-middle SDI | 65.80 | 70.03 | 61.72 | 1.13 | 69.34 | 73.41 | 64.34 | 1.14 | 37.43 | 39.33 | 36.10 | 1.09 | 25.32 | 25.86 | 25.00 | 1.03 |
|  | Low SDI | 65.75 | 73.06 | 58.32 | 1.25 | 64.01 | 71.58 | 54.00 | 1.33 | 29.12 | 28.65 | 29.39 | 0.97 | 23.24 | 23.20 | 23.27 | 1.00 |
| <1 year | Global | 80.79 | 84.96 | 76.05 | 1.12 | 81.57 | 84.47 | 77.80 | 1.09 | 51.13 | 59.70 | 45.78 | 1.30 | 34.85 | 39.55 | 32.09 | 1.23 |
|  | High SDI | 93.60 | 94.33 | 92.77 | 1.02 | 89.15 | 90.67 | 86.97 | 1.04 | 85.67 | 86.06 | 85.33 | 1.01 | 73.63 | 74.90 | 72.35 | 1.04 |
|  | High-middle SDI | 90.88 | 91.98 | 89.75 | 1.02 | 86.27 | 87.72 | 84.41 | 1.04 | 72.00 | 78.40 | 64.68 | 1.21 | 39.15 | 43.99 | 34.80 | 1.26 |
|  | Middle SDI | 86.55 | 88.05 | 85.12 | 1.03 | 84.22 | 86.06 | 82.01 | 1.05 | 61.87 | 70.45 | 53.27 | 1.32 | 32.51 | 37.27 | 28.91 | 1.29 |
|  | Low-middle SDI | 78.53 | 83.01 | 72.81 | 1.14 | 79.08 | 82.46 | 74.50 | 1.11 | 38.15 | 39.29 | 37.73 | 1.04 | 28.28 | 27.25 | 28.59 | 0.95 |
|  | Low SDI | 74.91 | 81.79 | 66.44 | 1.23 | 71.07 | 78.46 | 59.81 | 1.31 | 32.61 | 29.81 | 33.68 | 0.89 | 27.48 | 25.83 | 28.07 | 0.92 |
| 1-4 years | Global | 79.42 | 83.10 | 75.92 | 1.09 | 77.68 | 80.54 | 73.99 | 1.09 | 59.92 | 64.83 | 55.58 | 1.17 | 36.85 | 39.65 | 34.68 | 1.14 |
|  | High SDI | 93.29 | 93.95 | 92.65 | 1.01 | 86.96 | 88.96 | 84.69 | 1.05 | 86.19 | 86.61 | 85.84 | 1.01 | 74.61 | 75.87 | 73.47 | 1.03 |
|  | High-middle SDI | 90.04 | 90.92 | 89.16 | 1.02 | 82.94 | 84.41 | 81.15 | 1.04 | 74.77 | 78.58 | 70.34 | 1.12 | 41.01 | 43.80 | 38.40 | 1.14 |
|  | Middle SDI | 83.62 | 85.01 | 82.46 | 1.03 | 81.80 | 83.47 | 79.72 | 1.05 | 65.57 | 70.79 | 59.61 | 1.19 | 33.72 | 36.12 | 31.67 | 1.14 |
|  | Low-middle SDI | 73.96 | 78.49 | 69.22 | 1.13 | 72.84 | 76.60 | 67.26 | 1.14 | 42.90 | 44.43 | 41.80 | 1.06 | 24.08 | 24.41 | 23.86 | 1.02 |
|  | Low SDI | 66.85 | 75.88 | 57.70 | 1.32 | 63.25 | 71.43 | 51.94 | 1.38 | 30.86 | 31.22 | 30.66 | 1.02 | 20.87 | 21.32 | 20.64 | 1.03 |
| 5-9 years | Global | 76.02 | 78.59 | 73.92 | 1.06 | 74.47 | 78.14 | 70.91 | 1.10 | 51.31 | 55.36 | 47.79 | 1.16 | 34.78 | 37.53 | 32.62 | 1.15 |
|  | High SDI | 92.72 | 93.38 | 92.19 | 1.01 | 84.08 | 86.78 | 81.57 | 1.06 | 76.44 | 76.86 | 76.09 | 1.01 | 63.38 | 64.34 | 62.52 | 1.03 |
|  | High-middle SDI | 86.43 | 88.53 | 84.80 | 1.04 | 77.18 | 80.58 | 74.18 | 1.09 | 63.65 | 68.36 | 59.24 | 1.15 | 35.20 | 38.06 | 32.78 | 1.16 |
|  | Middle SDI | 78.99 | 81.82 | 76.76 | 1.07 | 77.47 | 81.28 | 73.69 | 1.10 | 53.67 | 58.88 | 48.52 | 1.21 | 30.13 | 32.99 | 27.83 | 1.19 |
|  | Low-middle SDI | 62.42 | 66.26 | 59.06 | 1.12 | 64.80 | 69.28 | 60.18 | 1.15 | 30.86 | 33.28 | 29.01 | 1.15 | 23.09 | 24.42 | 22.20 | 1.10 |
|  | Low SDI | 58.42 | 62.48 | 54.73 | 1.14 | 56.93 | 60.97 | 52.76 | 1.16 | 24.03 | 24.72 | 23.55 | 1.05 | 22.58 | 23.43 | 22.06 | 1.06 |
| 10-14 years | Global | 65.35 | 66.08 | 64.63 | 1.02 | 64.52 | 64.76 | 64.03 | 1.01 | 56.05 | 60.01 | 52.44 | 1.14 | 36.28 | 39.67 | 33.46 | 1.19 |
|  | High SDI | 83.71 | 84.41 | 83.12 | 1.02 | 74.53 | 76.58 | 72.70 | 1.05 | 81.72 | 82.51 | 81.09 | 1.02 | 68.86 | 70.53 | 67.41 | 1.05 |
|  | High-middle SDI | 76.90 | 77.93 | 76.03 | 1.03 | 66.97 | 67.35 | 66.42 | 1.01 | 69.06 | 73.28 | 64.94 | 1.13 | 36.47 | 40.58 | 33.00 | 1.23 |
|  | Middle SDI | 68.01 | 68.42 | 67.50 | 1.01 | 66.52 | 66.39 | 66.26 | 1.00 | 58.52 | 63.68 | 53.14 | 1.20 | 27.42 | 31.31 | 24.03 | 1.30 |
|  | Low-middle SDI | 52.06 | 52.86 | 51.19 | 1.03 | 55.30 | 55.42 | 54.85 | 1.01 | 27.98 | 31.19 | 25.37 | 1.23 | 15.08 | 17.04 | 13.60 | 1.25 |
|  | Low SDI | 46.87 | 47.97 | 45.76 | 1.05 | 44.61 | 45.00 | 44.03 | 1.02 | 16.68 | 17.50 | 16.06 | 1.09 | 11.68 | 12.24 | 11.31 | 1.08 |
| 15-19 years | Global | 64.15 | 65.66 | 63.03 | 1.04 | 58.21 | 59.77 | 57.00 | 1.05 | 63.15 | 65.95 | 60.74 | 1.09 | 50.94 | 52.81 | 49.52 | 1.07 |
|  | High SDI | 83.73 | 83.97 | 83.56 | 1.00 | 71.24 | 75.45 | 67.85 | 1.11 | 80.45 | 81.80 | 79.42 | 1.03 | 69.93 | 71.99 | 68.38 | 1.05 |
|  | High-middle SDI | 74.13 | 75.79 | 72.95 | 1.04 | 58.07 | 60.02 | 56.75 | 1.06 | 71.70 | 75.77 | 67.94 | 1.12 | 50.58 | 53.34 | 48.57 | 1.10 |
|  | Middle SDI | 61.82 | 64.15 | 60.15 | 1.07 | 56.67 | 57.46 | 56.05 | 1.03 | 63.57 | 67.49 | 60.01 | 1.12 | 45.82 | 47.75 | 44.30 | 1.08 |
|  | Low-middle SDI | 53.41 | 54.99 | 52.05 | 1.06 | 54.04 | 54.90 | 53.27 | 1.03 | 46.77 | 47.91 | 45.79 | 1.05 | 41.14 | 41.85 | 40.57 | 1.03 |
|  | Low SDI | 52.07 | 54.16 | 50.63 | 1.07 | 50.91 | 52.45 | 49.76 | 1.05 | 41.51 | 41.64 | 41.42 | 1.01 | 39.48 | 39.72 | 39.32 | 1.01 |


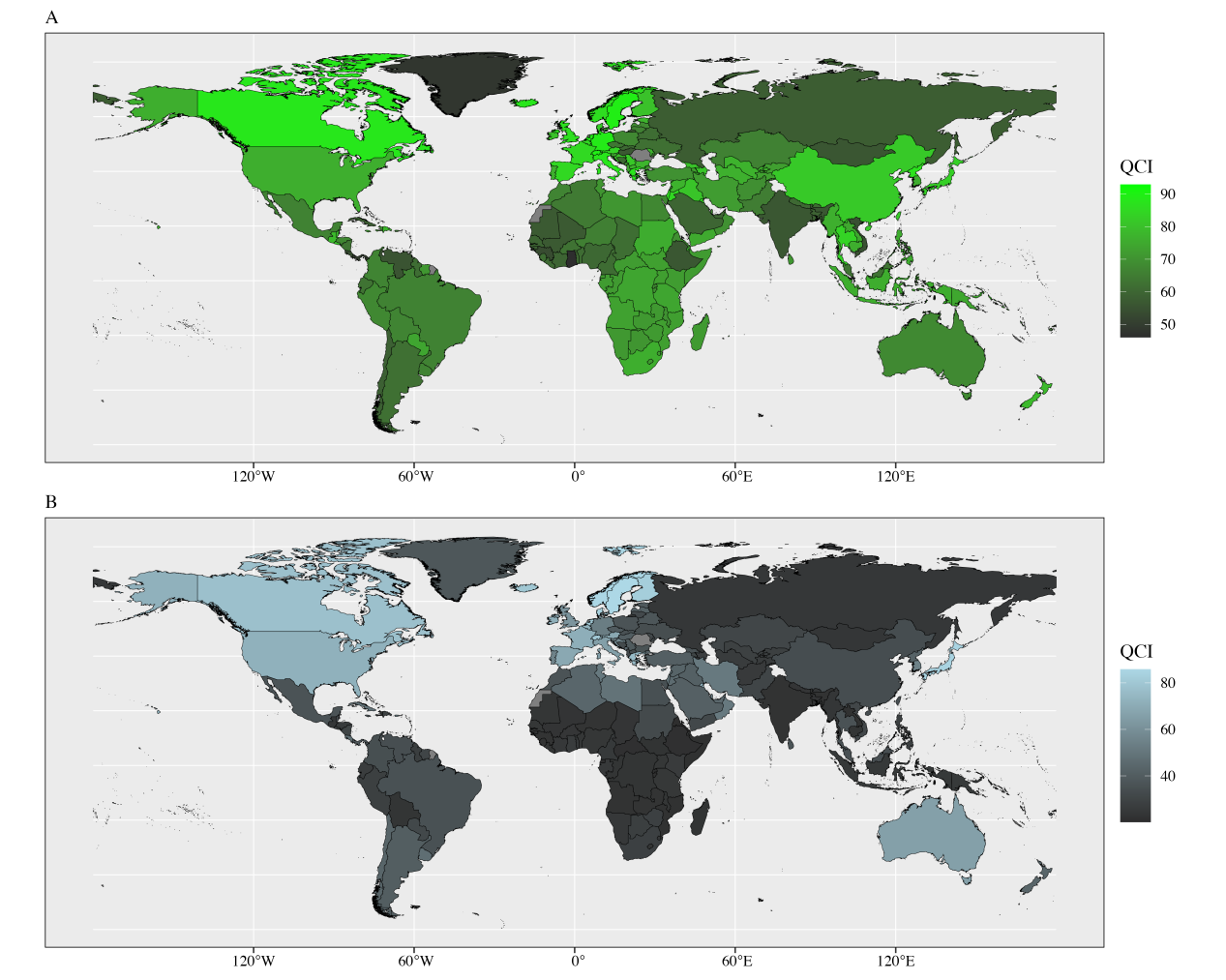


**Figure 1.** Global map of Quality of Care Index (QCI) for 2 childhood cancers in 1990. **Panel A.** Global map of Quality of Care Index (QCI) for Leukemia in 1990. **Panel B.** Global map of Quality of Care Index (QCI) for Brain and central nervous system (CNS) tumor in 1990.





**Figure 2.** Trend of Quality of Care Index (QCI) for two different types of childhood cancer from 1990 to 2019, by sociodemographic index (SDI) regions. **Panel A.** 30 years trend of QCI for Leukemia by SDI regions. **Panel B.** 30 years trend of QCI for Brain and central nervous system cancer by SDI regions. **Panel C.** 30 years trend of QCI for sum of 3 childrhood cancers by SDI regions.





**Figure 3.** QCI of childhood cancer in different age groups in 2019

**Panel A.** Leukemia. **Panel B.** Brain and central nervous system cancer. **Panel C.** Sum of 2 childrhood cancers.
