# Supplementary material for: Global, regional and national burden and quality of care index (QCI) of leukaemia and brain and central nervous system tumours in children and adolescents aged 0–19 years: a systematic analysis of the Global Burden of Disease Study 1990–2019
Source: BMJ Open. 2025 Mar 22;15(3):e093397. doi: 10.1136/bmjopen-2024-093397 (PMC11931942; doi:10.1136/bmjopen-2024-093397)
Supplement: Supplementary file 2 [file bmjopen-2024-093397.reviewer_comments.pdf]

PEER REVIEW HISTORY

BMJ Open publishes all reviews undertaken for accepted manuscripts. Reviewers are asked to complete a checklist review form and are provided with free text boxes to elaborate on their assessment. These free text comments are reproduced below.

ARTICLE DETAILS

Title (Provisional)

Global, regional, and national burden and quality of care index (QCI) of leukemia and brain and central nervous system tumors in children and adolescents aged 0-19 years: A systematic analysis of the Global Burden of Disease Study 1990-2019

Authors

Bi, Yanxin; Huang, Kepei; Wang, Minmin; Jin, Yinzi; Zheng, Zhi-Jie

---

VERSION 1 - REVIEW

**Reviewer**	**1**
**Name**	**Wakeford, Richard**
**Affiliation**	
**Date**	**22-Oct-2024**
**COI**	**I am a member of the Technical Working Party of the UK Compensation Scheme for Radiation-Linked Diseases (http://www.csrld.org.uk/) although I don't believe this is a real conflict of interest in respect of this manuscript.**

Please consider carefully my comments in the accompanying annotated manuscript because I think that certain parts of the manuscript need to be amended to make them clearer or to correct erroneous statements.

The statistical methods used in the study need to be explained in greater detail because at the moment these are unclear, especially with respect to interval estimation.

**** The reviewer provided a marked copy with additional comments. Please contact the publisher for full details. ****

**Reviewer**	**2**
**Name**	**Uwishema, Olivier**
**Affiliation**	**Oli Health Magazine Organization Kigali**
**Date**	**18-Dec-2024**

COI **None**

To the Authors:

Dear Author(s),

Thank you so much for giving me the opportunity to review the manuscript titled " **Global, regional, and national survey on burden and quality of care index (QCI) of childhood cancer: A systematic analysis of the Global Burden of Disease Study 1990-2019** ". While the study is well-conducted and offers significant value to the field, some improvements are needed. **I recommend major revision**.

Below are the comments to the authors. Once these comments are carefully and clearly addressed, the paper should be suitable for publication.

1. **Introduction:**
   - The introduction effectively sets the stage by highlighting the significance of childhood cancer and the WHO initiative. However, it lacks a clear statement on the novelty of the study. Consider specifying how your analysis on QCI contributes uniquely to the field.
2. **Methods:**
   - The methodology section is quite sparse. There's a need for a more detailed description of how data were selected, processed, and analyzed. Specifically:
     - Clarify the exact GBD data years used since the title mentions 1990-2019 but the text does not detail this.
     - Explain the choice and calculation of the QCI components more explicitly, including the rationale behind using PCA.
     - Describe the statistical analyses in more depth, particularly how trends were examined and how the EAPC was calculated.
3. **Results:**
   - Results are presented but could be organized better. For example, consider grouping findings by type of cancer (**leukemia** vs. **CNS tumors**) for clarity.
   - Some figures and tables are not very clear. Ensure all referenced figures and tables are very clear in the manuscript.
   - The discussion on gender and age trends needs more depth; currently, it's brief and lacks elaboration on why such trends might occur.
4. **Incorporating the published articles related to the topic:**

By grouping finding based on the type of cancer, consider citing this published paper on CNS tumors: Uwishema, O., Frederiksen, K. S., Badri, R., Pradhan, A. U., Shariff, S., Adanur, I., Dost, B., Esene, I., & Rosseau, G. (2023). Epidemiology and etiology of brain cancer in Africa: A systematic review. *Brain and behavior*, *13*(9), e3112. https://doi.org/10.1002/brb3.3112

5. **Discussion:**
   - The discussion does a good job linking findings with existing literature but could be stronger in suggesting implications for policy or practice based on these findings.
   - Address potential confounders or biases that might affect your results, especially concerning the interpretation of gender disparities in QCI.
6. **Conclusion:**
   - The conclusion should more directly address the implications of the findings for health policy or clinical practice, particularly in low and middle-income countries where disparities are most pronounced.
7. **General Comments:**
   - Proofread for grammatical errors and improve sentence structure in several places for better readability.
   - Consider adding a limitations section before the conclusion to discuss data constraints or methodological limitations in more detail.

VERSION 1 - AUTHOR RESPONSE

Reviewer #1:

1. Please consider carefully my comments in the accompanying annotated manuscript because I think that certain parts of the manuscript need to be amended to make them clearer or to correct erroneous statements.

Authors' response: Thank you for your thoughtful and detailed comments on the manuscript. I have carefully reviewed the annotated manuscript and made the necessary revisions to address the concerns you raised.

a. Thank you for your careful review and valuable feedback on the manuscript. I have thoroughly reviewed the annotated manuscript and made revisions to address the grammar and expression errors you pointed out. Your comments helped identify areas where clarity could be improved, and I have made sure to correct these issues to enhance the overall readability and accuracy of the text. I truly appreciate your input, as it has been instrumental in refining the manuscript.

b. The reviewer pointed out in the first paragraph of the Introduction (line 4, marked as "19") that it might be useful to briefly explain the change from 0-14 years of age to 0-19 years of age. In response to this, we have revised the manuscript on **page 5, lines 4-9**, to clarify that the age range of 0-19 years is in line with the World Health Organization's definition of childhood, which includes both children (0-14 years) and adolescents (15-19 years). This broader age range is commonly used in global reports to provide a comprehensive view of childhood cancer incidence across all age groups.

c. The reviewer raised a valid concern regarding the completeness of childhood cancer registration across countries, especially those with varying socio-economic conditions.
In response, we have expanded the limitations section to address this issue. We acknowledged that the completeness and accuracy of childhood cancer data can differ by region, with more complete data available in countries with better cancer registries, and less reliable data in lower-income countries due to challenges like underreporting and limited resources. This explanation has been incorporated to help readers interpret the data from the GBD dataset in context.

d. Thank you for your comment regarding the calculation of the 95% confidence interval (CI) for the Estimated Average Percentage Change (EAPC).
To clarify, the EAPC is derived from the generalized linear model by fitting the natural logarithm of the age-standardized rate (ASR) to the calendar year. The EAPC is calculated as $100\times(\exp[\beta]-1)$, where β represents the rate of change over time. The 95% CI for the EAPC is obtained from the regression model by applying the exponential transformation to the CI of β. As for including further details in the manuscript, we believe the current explanation is sufficient for the readers to understand the methodology. We elaborate on this section in detail on page 8, lines 11-18 of the manuscript.

e. Thank you for your comment regarding the calculation of the 95% uncertainty interval (UI) for the main indices.
The uncertainty interval (UI) is provided by the Global Burden of Disease (GBD) study as part of their estimates, which incorporate a range of uncertainty due to data limitations, model assumptions, and other factors. The UI is calculated using a probabilistic approach, where multiple simulations are run to reflect the uncertainty in the input data and the modeling

process. This results in an interval that represents the range within which the true value is likely to lie, with a certain level of confidence.

In contrast, the confidence interval (CI) reflects the uncertainty associated with a sample estimate in a given study. While both the CI and UI reflect uncertainty, the CI is typically derived from the statistical model applied to the sample data, whereas the UI reflects a broader range of uncertainty, accounting for both data and model uncertainties.

We believe that the current explanation is sufficient and aligns with the standard practices of the GBD methodology. Let me know if you'd like any further changes!

f. Thank you for your insightful comment regarding the statement on statistical significance. We appreciate your suggestion to refine the approach for assessing differences between the comparison groups.

We fully acknowledge that comparing the overlap of uncertainty intervals (UIs) may be perceived as a somewhat crude method. However, this approach was used as a general guideline to assess the significance of differences in the absence of more advanced statistical tests. Importantly, we relied on the uncertainty intervals (UIs) provided by the Global Burden of Disease (GBD) study, which are publicly available data. These UIs are an integral part of the GBD methodology and are designed to quantify the uncertainty in the estimates. As we did not perform independent calculations for the UIs but used the data as provided by GBD, we believe that the method aligns with the standard practices of the GBD study.

For your reference, the official GBD methodology and the corresponding uncertainty intervals can be found in the following publication:

Global Burden of Disease Study 2019: Estimating Global, Regional, and National Incidence, Prevalence, and Years Lived with Disability for 358 Diseases and Injuries for 204 Countries and Territories, 1990-2019: A Systematic Analysis for the Global Burden of Disease Study 2019 Murray, C. J. L., et al. The Lancet, 2020, 396(10258), 1200-1222.

Once again, thank you for your valuable feedback. Please feel free to let us know if you have any further questions.

g. Thank you for your suggestion regarding the uncertainty intervals (UIs) for the number of deaths.

We greatly appreciate this suggestion. In response, we have removed the UIs for the number of deaths from the Results section. We now focus on the age-standardized mortality rates and their

associated UIs, which more accurately reflect the temporal trends and underlying disease burden. See page 10, lines 6-9 for details.

h. We appreciate your observation regarding the terms "increased" and "decreasing trend".
We greatly appreciate this constructive comment. In this section, we aimed to provide a descriptive overview of the changes in the proportions of childhood cancer deaths over time without conducting statistical tests for significance. Our focus was on highlighting the general trends in these proportions rather than drawing conclusions about their statistical significance. We agree that further analysis could be valuable, but for the purpose of this study, we chose to present these trends descriptively.

i. We appreciate your observation regarding the negative EAPC for childhood cancer.
We sincerely thank you for your insightful comment regarding the negative EAPC for childhood cancer. You are absolutely correct that a negative EAPC indicates a decreasing trend. In response to your feedback, we have revised the manuscript in four places to ensure greater clarity. Specifically, the following changes were made: "while Central Latin America had the largest decreasing trend of the QCI index for leukemia, with an EAPC of -0.41 (95% CI: -0.45, -0.38)" (Page 11, lines 9-10); "while Oceania had the largest decreasing trend of the QCI index, with an EAPC of -0.03 (95% CI: -0.11, 0.05) from 1990 to 2019" (Page 11, lines 12-13); "Kyrgyzstan showed the largest decreasing trend of the QCI index (EAPC=-0.77, 95% CI: -0.84, -0.71) for leukemia" (Page 11, lines 20-21); and "Zimbabwe showed the largest decreasing trend of the QCI index (EAPC=-0.99, 95% CI: -1.20, -0.78) for brain and CNS tumors" (Page 11, lines 22 to Page 12, lines 1-2). We are very grateful for your help in enhancing the clarity of our manuscript.

j. Thank you for your valuable feedback on our manuscript. You raised an important point regarding the interpretation of the EAPC for Oceania in the results section. We appreciate your careful review and agree that the confidence interval for the EAPC in Oceania includes zero, indicating that the trend is not statistically significant.
To address this concern, we have revised the sentence to focus on the regions with the highest and lowest significant trends. The updated sentence now reads: "For CNS tumors, East Asia showed the highest growing trend of QCI index with an EAPC of 2.93 (95% CI: 2.78, 3.08), while Central Sub-Saharan Africa showed the smallest increase with an EAPC of 0.12 (95%

CI: 0.06, 0.19) from 1990 to 2019 (Supplementary Table 2)." This change can be found on page 10, lines 22 to page 11, lines 1-2 of the revised manuscript.

We believe this revision provides a clearer and more focused interpretation of the data. Thank you again for your insightful comments, which have helped improve the clarity and accuracy of our manuscript..

2. The statistical methods used in the study need to be explained in greater detail because at the moment these are unclear, especially with respect to interval estimation.

Authors' response:

Thank you for your valuable comments regarding the statistical methods used in this study. We appreciate your suggestion to provide a more detailed explanation, particularly regarding interval estimation, and we have revised the statistical analysis section accordingly to ensure greater clarity.

Related revisions were made in the manuscript as "For this analysis, a generalized linear regression model was applied to the natural logarithm of age-standardized ratios (ASR) for QCI, YLLs, YLDs, and other relevant metrics. The regression model took the form $\ln(ASR)=\alpha+\beta\cdot Year+\varepsilon$, where α represents the intercept, β represents the positive or negative ASR trends, and ε is the error term. The EAPC was derived using the formula $100\times(\exp[\beta]-1)$ and its 95% CIs were obtained directly from the regression coefficients. A positive EAPC value indicated an increasing trend, whereas a negative EAPC signified a decreasing trend over the study period. " (Page 8, Line 11-18)

Additionally, we expanded on the statistical approach used to examine the correlation between QCI and the Sociodemographic Index (SDI). We calculated Pearson correlation coefficients to investigate whether higher socioeconomic development levels were associated with better quality of care. The SDI is a composite indicator that reflects a country's income per capita, average years of schooling, and fertility rate among women under 25 years of age. We have also described how we investigated disparities in QCI across sex and age groups. For gender disparity, we calculated the gender disparity ratio (GDR) as the ratio of QCI in girls divided by QCI in boys, where a GDR greater than 1 indicates better QCI in girls. We also analyzed QCI scores across different age groups (<1 year, 1-4 years, 5-9 years, 10-14 years, and 15-19 years) to identify trends related to age. (Page 9, Line 2-16)

Finally, we have clarified the calculation of uncertainty intervals (UIs) for all GBD-derived estimates. UIs were computed following standardized GBD methods, which take into account

sampling variability, non-sampling error, and model uncertainty. These intervals were used to assess statistical significance, which was defined as non-overlapping UIs between comparison groups. (Page 9, Line 17-20)

Reviewer #2:

Major Comments:

1. Introduction:

- The introduction effectively sets the stage by highlighting the significance of childhood cancer and the WHO initiative. However, it lacks a clear statement on the novelty of the study. Consider specifying how your analysis on QCI contributes uniquely to the field.

Authors' response: Thank you for your valuable feedback regarding the novelty of the study in the Introduction section. We appreciate your insight and have revised the introduction to clearly highlight the unique contribution of our analysis on the Quality of Care Index (QCI) in the context of childhood cancer.

In response to your suggestion, we have emphasized how our study contributes uniquely to the field by introducing a comprehensive and systematic approach to evaluating the quality of care for childhood cancer at global, regional, and national levels. While previous studies have linked QCI with cancer prognosis and disease burden, our study extends this understanding by evaluating QCI disparities across different regions, income levels, gender, and age groups. We believe this multifaceted analysis is crucial to understanding the complex factors that influence childhood cancer care quality and will provide essential evidence to identify vulnerable populations. This, in turn, will help inform more targeted interventions to improve childhood cancer outcomes, particularly in underserved regions and among at-risk groups.

We trust that this revision clarifies the novel aspects of our study and underscores its significance in advancing global efforts to reduce childhood cancer burden and enhance care quality.

2. Methods:

- The methodology section is quite sparse. There's a need for a more detailed description of how data were selected, processed, and analyzed. Specifically:
- Clarify the exact GBD data years used since the title mentions 1990-2019 but the text does not detail this.
- Explain the choice and calculation of the QCI components more explicitly, including the rationale behind using PCA.
- Describe the statistical analyses in more depth, particularly how trends were examined and how the EAPC was calculated.

Authors' response:

We sincerely thank the reviewers for their constructive feedback on our methodology section. We have carefully addressed each of the concerns raised and have revised the manuscript

accordingly to provide greater clarity and detail. Below, we provide a point-by-point response to the reviewers' comments.

Regarding the clarification of the exact GBD data years used:

We appreciate the reviewer's observation regarding the need for explicit mention of the GBD data years. In the revised manuscript, we have clearly stated that the data were retrieved from the Global Burden of Diseases (GBD) 2019 dataset, which spans from 1990 to 2019 and covers 204 countries and territories. This clarification ensures alignment with the title and the scope of our study. See Page 6, Line 22 for details.

Regarding the explanation of the choice and calculation of the QCI components, including the rationale behind using PCA:

We thank the reviewer for highlighting the need for a more explicit explanation of the Quality of Care Index (QCI) components and the use of Principal Component Analysis (PCA). In the revised manuscript, we have elaborated on the selection of the four secondary indicators used to construct the QCI: the ratio of years of life lost (YLLs) to years lived with disability (YLDs), the DALYs to prevalence ratio, the mortality to incidence ratio (MIR), and the prevalence to incidence ratio. These indicators were chosen based on their established relevance in assessing healthcare outcomes for childhood cancers and their use in prior studies (Hanifiha et al., 2022; Mohammadi et al., 2021; Keykhaei et al., 2021).

PCA was employed to address the multicollinearity among these indicators and to reduce the dimensionality of the data, ensuring that the resulting index captures the maximum variance in the dataset. The first principal component extracted from PCA was scaled to a 0-100 range to derive the final QCI score, where higher values indicate better quality of care. This approach aligns with best practices in index construction, as it provides a robust and interpretable measure of care quality while minimizing redundancy among the components (Mohammadi et al., 2021; Keykhaei et al., 2021). See Page 7, Line 16-22 and Page 8, Line 1-7 for further details.

Regarding the description of the statistical analyses, particularly the examination of trends and calculation of the EAPC:

We appreciate the reviewer' s request for a more detailed explanation of the statistical analyses. In the revised manuscript, we have expanded the description of how the Estimated

Annual Percentage Change (EAPC) was calculated. Specifically, we applied a generalized linear regression model to the natural logarithm of age-standardized ratios (ASR) for QCI, YLLs, YLDs, and other relevant metrics. The regression model took the form $\ln(ASR) = \alpha + \beta \cdot Year + \varepsilon$, where α represents the intercept, β represents the positive or negative ASR trends, and ε is the error term. The EAPC was derived using the formula $100\times(\exp[\beta]-1)$, and its 95% confidence intervals (CIs) were obtained directly from the regression coefficients. A positive EAPC value indicates an increasing trend, whereas a negative EAPC signifies a decreasing trend over the study period. This method ensures a robust assessment of trends over time and their statistical significance. See Page 8, Line 11-18 for additional information.

Regarding the explanation of the Sociodemographic Index (SDI):

We have also ensured that the Sociodemographic Index (SDI) is adequately explained in the revised manuscript. The SDI is a composite measure that reflects a country's level of lag-distributed income per capita, average years of schooling, and fertility rate in females under the age of 25 years (GBD Collaborators, 2020). It was used to explore associations between socioeconomic status and the quality of care, providing valuable insights into disparities across regions. In 2019, countries and regions were categorized into five SDI levels: high (0.81-1.00), high-middle (0.70-0.81), middle (0.61-0.69), low-middle (0.46-0.60), and low (0.00-0.45) (GBD Collaborators, 2021). See Page 9, Line 2-4 for additional information. Additionally, disparities in QCI across sex and age groups were investigated using the gender disparity ratio (GDR) and age-specific analyses. See Page 9, Line 11-16 for further elaboration.

We believe that these revisions have significantly enhanced the clarity and depth of the methodology section. We are grateful for the reviewers' insightful comments, which have helped us improve the manuscript. Thank you once again for your valuable feedback.

3. Results:

- Results are presented but could be organized better. For example, consider grouping findings by type of cancer (leukemia vs. CNS tumors) for clarity.
- Some figures and tables are not very clear. Ensure all referenced figures and tables are very clear in the manuscript.
- The discussion on gender and age trends needs more depth; currently, it's brief and lacks elaboration on why such trends might occur.

Authors’ response:

Thank you for your insightful comments and suggestions. We have carefully revised the Results section in response to your feedback:

Regarding the organization of findings by type of cancer:

We have reorganized the Results section to present findings separately for leukemia and CNS tumors, as per your suggestion. This grouping enhances clarity and allows for a more direct comparison between the two cancer types.

Regarding the clarity of figures and tables:

We have reviewed all referenced figures and tables to ensure they are clear and properly labeled. We have made necessary adjustments to improve their readability and clarity.

Regarding the discussion of gender and age trends:

Thank you for your insightful comments regarding the in-depth discussion of gender and age trends. We appreciate your suggestion to elaborate on these aspects within the Results section. However, we would like to clarify that the Results section primarily focuses on presenting the findings, while the detailed discussion of potential causes has already been addressed in the Discussion section of the manuscript. In particular, we have provided a comprehensive analysis of gender and age disparities in the Quality of Care Index (QCI) for childhood cancer.

In this section, we discuss the higher QCI for girls compared to boys, which may be related to the higher burden and worse prognosis of childhood cancer in boys. Genotypic and biological differences in childhood cancer, including the higher incidence of certain leukemia subtypes like acute myeloid leukemia (AML) and chronic lymphocytic leukemia (CLL) in boys, contribute to this disparity. We also acknowledge that cultural biases may lead to underdiagnosis in girls in some regions. Additionally, the increasing gender disparity in QCI for brain and CNS tumors is explored, with boys having a higher incidence of malignant tumors and girls showing a higher incidence of non-malignant types. This variation in tumor subtype distribution is likely a key factor in the growing gender disparity in QCI over time. Furthermore, we examined how the QCI for boys and girls varies across regions with different SDI levels, with girls receiving higher care quality in high SDI regions and a more balanced care quality in those areas. We hope this explanation clarifies that the detailed discussions regarding the potential reasons for gender and age disparities in QCI have been appropriately placed in the Discussion section, where we delve deeper into these factors. We appreciate your

careful consideration of our manuscript and trust that these clarifications address your concerns.

4. Incorporating the published articles related to the topic:
By grouping finding based on the type of cancer, consider citing this published paper on CNS tumors: Uwishema, O., Frederiksen, K. S., Badri, R., Pradhan, A. U., Shariff, S., Adanur, I., Dost, B., Esene, I., & Rosseau, G. (2023). Epidemiology and etiology of brain cancer in Africa: A systematic review. Brain and behavior, 13(9), e3112. https://doi.org/10.1002/brb3.3112

Authors' response:

We sincerely thank the reviewer for their valuable suggestion to incorporate the article by Uwishema et al. (2023) into our discussion. This paper provides a comprehensive review of the epidemiology and etiology of CNS tumors in Africa, which complements our study's global perspective on childhood CNS tumors. While their focus is on the African context, the findings underscore the significant challenges faced in low-resource settings, such as limited access to diagnostic and treatment facilities, which are consistent with our observations of disparities in care quality across different sociodemographic levels. We have integrated this reference into the Discussion section to highlight the regional challenges in CNS tumor care and to emphasize the need for targeted interventions in under-resourced regions. We believe this addition strengthens the relevance and depth of our discussion. Thank you for this insightful suggestion. (Page 15, Lines 10-15)

5. Discussion:
o The discussion does a good job linking findings with existing literature but could be stronger in suggesting implications for policy or practice based on these findings.
o Address potential confounders or biases that might affect your results, especially concerning the interpretation of gender disparities in QCI.

Authors' response:

We thank the reviewer for the valuable feedback. In response to the first comment regarding the need to strengthen the implications for policy and practice, we have revised the discussion to emphasize the importance of considering gender as a key factor in the design of childhood cancer care programs. This is particularly crucial in low- and middle-income countries (LMICs), where disparities in access to care are often exacerbated by socio-economic and cultural factors. We have added the following text to Page 18, Lines 17-22, to Page 19, Lines 1-2:

"To address this issue, it is crucial for policymakers to consider gender as a factor in the design of childhood cancer care programs, particularly in low- and middle-income countries (LMICs), where disparities in care access are often exacerbated by socio-economic and cultural factors. Promoting gender-sensitive healthcare interventions and ensuring equal access to diagnosis, treatment, and supportive care for both sexes should be prioritized in national cancer strategies. Additionally, further research is needed to explore the underlying causes of these disparities and develop targeted interventions."

Regarding the second comment on potential confounders or biases affecting the results, particularly concerning gender disparities in QCI, we have acknowledged that factors such as differences in healthcare access, socioeconomic status, and regional healthcare policies may influence these results. These factors should be carefully considered when interpreting the findings. The following text has been added to page 19, lines 17–20:

"However, potential confounders such as differences in healthcare access, socioeconomic status, and regional healthcare policies may influence these results and should be considered when interpreting the gender disparities in QCI."

We believe these revisions provide a more comprehensive discussion of the implications of our findings for policy and practice, and address the potential confounders that could affect the interpretation of gender disparities in QCI.

6. Conclusion:

The conclusion should more directly address the implications of the findings for health policy or clinical practice, particularly in low and middle-income countries where disparities are most pronounced.

Authors' response:

We sincerely thank the reviewer for the valuable feedback. In response to the comment, we have revised the conclusion to more directly address the implications of our findings for health policy and clinical practice, particularly in low- and middle-income countries (LMICs) where disparities in access to care are most pronounced. We emphasize the need for targeted interventions, gender-sensitive healthcare strategies, and improving access to diagnosis, treatment, and supportive care in these regions.

The following sentence has been added to the conclusion:

"Countries with low social development levels and boys should be prioritized in policy interventions to reduce health disparities. To address these issues, policymakers in low- and middle-income countries (LMICs) should focus on improving access to quality care,

particularly for boys and populations in lower SDI regions. Ensuring equitable healthcare policies, increasing access to early diagnosis, and enhancing treatment options will be crucial in addressing these disparities."

This addition can be found on page 21, lines 20-22, to page 22, lines 1-6. We believe this revision strengthens the conclusion by explicitly highlighting the practical implications for health policy and clinical practice, as suggested by the reviewer.

7. General Comments:

o Proofread for grammatical errors and improve sentence structure in several places for better readability.

o Consider adding a limitations section before the conclusion to discuss data constraints or methodological limitations in more detail.

Authors' response: We greatly appreciate the reviewer's insightful comments regarding both the grammatical issues and the limitations of our study. Regarding the grammatical errors, both you and Reviewer 1 pointed them out, and I have carefully reviewed and revised the manuscript accordingly. The sentence structure has been refined, and grammatical errors have been corrected to improve the overall readability and flow of the text.

Additionally, in response to the reviewer's comment on the limitations section, we have made several revisions to more thoroughly address data constraints and methodological limitations, particularly concerning the IHME-GBD dataset. We have expanded the limitations section to include more details on potential reporting biases, incomplete data, and the varying standards of data collection across countries. These factors may impact the accuracy and completeness of the dataset. We have also clarified that, due to data unavailability, we were unable to assess disease subtypes and ethnicity separately. Furthermore, we highlighted the variation in cancer registration completeness across countries with different socio-economic statuses, which may contribute to discrepancies in the dataset. Finally, we have emphasized the limitations of our cross-sectional study design, noting that causal relationships cannot be drawn from the data.

These revisions are incorporated in the "limitations" section on pages 20, lines 21-22, and page 21, lines 1-11. We believe these additions address the reviewer's concerns and provide a clearer explanation of the data constraints and methodology of the study.

Once again, we would like to thank the reviewer for their valuable feedback, which has helped us improve the rigor and clarity of our manuscript.

VERSION 2 - REVIEW

**Reviewer**	**1**
**Name**	**Wakeford, Richard**
**Affiliation**	
**Date**	**06-Mar-2025**
**COI**	

Thank you for addressing my comments on the original manuscript.

**Reviewer**	**2**
**Name**	**Uwishema, Olivier**
**Affiliation**	**Oli Health Magazine Organization Kigali**
**Date**	**14-Feb-2025**
**COI**	

Many thanks to the authors, and congratulations! All comments and suggestions from my end have been meticulously addressed and incorporated into the current version of the paper. Each critique was carefully evaluated, and corresponding modifications were made to enhance the scholarly quality, scientific rigor, and overall presentation of the research. The manuscript now meets academic standards and publication criteria. After a thorough assessment, I confidently recommend that this revised version is fully prepared and suitable for publication in this journal.
